# Autistic Traits in Neurotypical Adults: Correlates of Graph Theoretical Functional Network Topology and White Matter Anisotropy Patterns

**DOI:** 10.1371/journal.pone.0060982

**Published:** 2013-04-05

**Authors:** Andras Jakab, Miklos Emri, Tamas Spisak, Anita Szeman-Nagy, Monika Beres, Sandor Attila Kis, Peter Molnar, Ervin Berenyi

**Affiliations:** 1 Department of Biomedical Laboratory and Imaging Science, University of Debrecen Medical and Health Science Center, Debrecen, Hungary; 2 Nuclear Medicine Institute, University of Debrecen Medical and Health Science Center, Debrecen, Hungary; 3 Department of Personality- and Clinical Psychology, Psychology Institute, University of Debrecen, Debrecen, Hungary; 4 Department of Behavioral Sciences, University of Debrecen Medical and Health Science Center, Debrecen, Hungary; University of California San Diego, United States of America

## Abstract

Attempts to explicate the neural abnormalities behind autism spectrum disorders frequently revealed impaired brain connectivity, yet our knowledge is limited about the alterations linked with autistic traits in the non-clinical population. In our study, we aimed at exploring the neural correlates of dimensional autistic traits using a dual approach of diffusion tensor imaging (DTI) and graph theoretical analysis of resting state functional MRI data. Subjects were sampled from a public neuroimaging dataset of healthy volunteers. Inclusion criteria were adult age (age: 18–65), availability of DTI and resting state functional acquisitions and psychological evaluation including the Social Responsiveness Scale (SRS) and Autistic Spectrum Screening Questionnaire (ASSQ). The final subject cohort consisted of 127 neurotypicals. Global brain network structure was described by graph theoretical parameters: global and average local efficiency. Regional topology was characterized by degree and efficiency. We provided measurements for diffusion anisotropy. The association between autistic traits and the neuroimaging findings was studied using a general linear model analysis, controlling for the effects of age, gender and IQ profile. Significant negative correlation was found between the degree and efficiency of the right posterior cingulate cortex and autistic traits, measured by the combination of ASSQ and SRS scores. Autistic phenotype was associated with the decrease of whole-brain local efficiency. Reduction of diffusion anisotropy was found bilaterally in the temporal fusiform and parahippocampal gyri. Numerous models describe the autistic brain connectome to be dominated by reduced long-range connections and excessive short-range fibers. Our finding of decreased efficiency supports this hypothesis although the only prominent effect was seen in the posterior limbic lobe, which is known to act as a connector hub. The neural correlates of the autistic trait in neurotypicals showed only limited similarities to the reported findings in clinical populations with low functioning autism.

## Introduction

The American Psychiatric Association defines autism as a pervasive developmental disability with impairments in social interaction, verbal, nonverbal communication and limited behavioral flexibility [1]. Conventionally, if an individual with autism has an intelligence quotient in the normal range (or above), the term high-functioning autism (HFA) is used, while meeting all of the criteria for HFA except communicative abnormality or history of language delay defines Asperger's syndrome [2]. Neuroanatomic observations provided evidence about micro- and macrostructural abnormalities in the autistic brain [3] and in the quest for characterizing such malformations, neuroimaging gained prominent attention. MRI volumetry revealed abnormal developmental pattern in autism, which is characterized by an early brain overgrowth that diminished in adults [4], [5]. Further evidence on localized structural abnormalities was provided by voxel-based morphometry studies [6]–[8] and cortical thickness measurements [9], [10]. Distinct anatomic correlates were linked with Asperger's Syndrome [11] and high-functioning autism [12]. Technological developments in the last two decades enabled the use of more advanced structural and functional magnetic resonance approaches. Here we shortly summarize the contributions of diffusion magnetic resonance and functional MR (fMRI) to autism research.

Diffusion-weighted imaging (DWI) is based on the sensitization of the magnetic resonance signal to molecular displacements while diffusion tensor imaging (DTI) acquires information on the directional preference of the anisotropic diffusion in the brain [13]. The driving force behind DWI's 25-year success story is that opened a new window on the characterization of tissue microarchitecture by probing the microscopic, diffusion-driven molecular motions. This approach gave rise to diverse studies that elucidated the correlations between regional changes of diffusion anisotropy and various psychopathologies [14] or linked the diffusion characteristics with cognitive phenotypes [15]. Lowered quantitative measures, such as the DTI-derived diffusion anisotropy, were reported in the white matter of autistic children's brains [16], [17] or in adults with Asperger's syndrome [18]. Inconsistently, some studies report regionally increased fractional anisotropy (FA) in diverse brain regions [19], [20].

Functional magnetic resonance imaging (fMRI) is an optimal modality for the in vivo observation of the working brain and to infer patterns of brain activity from the changes of the blood oxygen-level dependent (BOLD) MR signal. In autism research putative functional impairments can be characterized by using experimental tasks such as executive function tests [21], cognitive control [22] or verbal fluency tasks [23]. The endeavor to study the normally functioning or pathologically altered macroscopic functional networks of the human brain was recently revitalized by the resting state fMRI concept [24]. In such studies the fundamental hypothesis is that the temporal coherence of BOLD signal time-courses marks the degree to which two regions are functionally connected [25], although this simple model does not imply causality and further inconsistency of such reports is caused by the large number of signal and image processing approaches that are in use [26]. More complex models can be used to examine the brain as a network of interconnected processing units rather than interpreting individually measured functional connections. One such mathematical approach is graph theory where brain regions define graph nodes and edges represent the strengths of structural or functional connectivity. This emerging network-based technique allows the calculation of graph topological measures that hallmark the properties of information exchange. The most commonly used indicators are small-worldness, efficiency, degree, modularity. Such quantitative studies are increasingly recognized – we refer to review articles on the background and possible clinical applications of the graph theoretical analysis of the human brain connectome [27], [28].

Resting state fMRI measurements, diffusion tensor imaging and tractography are potentially applicable to indirectly explore the disturbed neuronal communication in autism. Our study aims to stand in the line of studies that assume abnormal formation of cortico-cortical short or long-range interconnections. Such models postulate impaired long-range connectivity in autism and in HFA which is also hallmarked by decreased diffusion anisotropy that predominantly affects frontal, fronto-parietal and fronto-occipital pathways [29]. Conversely, a minority of studies revealed increased connectivity values or diffusion anisotropy [30], [31] particularly coinciding with the spatial patterns of short-range association fibers [19], [32]. The reported inconsistencies in neuroimaging studies reflect the puzzling nature of autism in which the core anatomical features and the common pathomechanisms of disrupted connectivity remains unknown.

Our investigation was designed to elucidate the putative effects of the autistic traits on the patterns of functional brain connectivity and diffusion anisotropy. Autism spectrum disorders are commonly understood to comprise traits with dimensionality; such traits can plausibly be quantified by clinical batteries or self-reported screening tests [33]. Accordingly, we assume that normally functioning adults with a certain degree of social reciprocity impairment and inflexible behavior present altered structural and functional brain connectivity. This supposition prompted us to employ a graph theoretical analysis that is presumably feasible and sensitive for characterizing changes in the functional topology of resting state networks (RSNs). We aim to relate the RSN topological correlates of autistic traits to the commonly reported observations from the broader spectrum of the disease.

## Methods

### Study population

Phenotypic information and imaging data of 207 subjects were taken from the public repository of the International Neuroimaging Data-sharing Initiative (INDI); we used the most recent release of the Nathan Kline Institute's Rockland Sample [34]. It is a freely available, large-scale, extensively phenotyped dataset for the discovery research and it contains healthy subjects from nearly all age groups. During sampling, inclusion criteria were: (i) age between 18 and 65, (ii) availability of the Wechsler Abbreviated Scale of Intelligence (WASI), Social Responsiveness Scale (SRS) and the autism spectrum screening questionnaire (ASSQ) scores, (iii) availability of DTI and resting-state fMRI scans, (iv) IQ above 70. As a result of this selection, we performed our analysis on a cohort of 127 neurotypical adults. The original NKI/Rockland data collection was preceded by the approval of the relevant ethical committee and subjects gave informed consent to the imaging studies.

### Psychological evaluations

**Figure 1:**
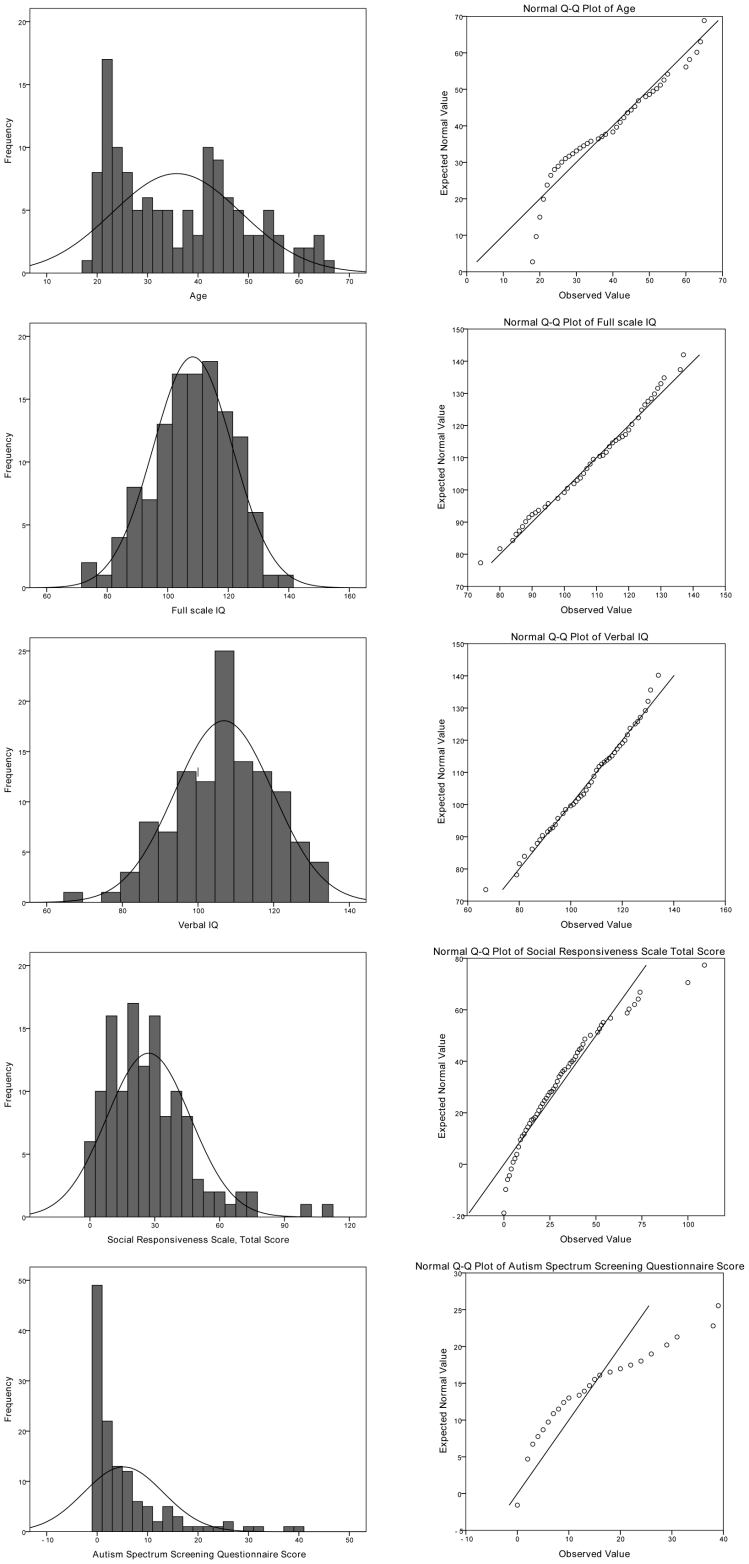
Demographic data of the study population. Distribution of subject age, full scale IQ, verbal IQ, ASSQ and SRS scores. Data are depicted as histograms (left panels) and Q-Q (quantile-quantile) probability plots (right panels) in which reference lines of the normal distribution are given (n = 127).

Our hypothesis is that inter-individual variability in dimensional autistic traits is correlated with characteristic changes in the functional network topology and diffusion anisotropy in neurotypical adults. The study was consequently designed to utilize multiple psychological tests as explanatory variables for functional topological measurements. First, we accessed data of the Autism Spectrum Screening Questionnaire (ASSQ), which is a 27 item checklist for completion by lay informants. This battery was reported to be plausible in assessing symptoms characteristic of Asperger's syndrome and other high functioning autism spectrum disorders in children and adolescents with normal intelligence [35]. The ASSQ was self-administered by the adults participating in this study. Data of self-reported autistic traits were completed by using the Social Responsiveness Scale (SRS) Adult version. The SRS is a 65 item questionnaire originally intended for an informant, but it was completed by the subjects here. Total SRS scores range from 0, corresponding to high social competence, to 195, corresponding to severe social impairment as observed in individuals with severe ASD. Scores between 60 and 80 are associated with mild forms of ASD [36]. SRS measures autistic traits that are continuously distributed in the normal population and has been used in similar investigations that correlate such traits with functional neuroimaging based data [37]. HFA is occasionally characterized by discrepant IQ profile, in other words, performance IQ scores significantly exceeding verbal IQ [38], but this observation is not ubiquitous [39]. To evaluate the intelligence profile, the authors of the NKI/Rockland dataset utilized the WASI, which consists of four subtests [40]. The performance IQ is composed of the scores of two subsets: the Block Design and Matrix reasoning, while the verbal IQ comprises the Vocabulary and Similarities tests. The distribution of the above mentioned demographic variables were tested for normality with one sample Kolmogorov-Smirnov (K-S) procedure. WASI verbal and full IQ scores were normally distributed (K-S; p = 0.57, 0.81). Age was not found to be normally distributed due to the lower and upper thresholds applied (K-S; asymptotic significance; p = 0.031). SRS total score was normally distributed (K-S; p = 0.23) while ASSQ showed significant deviation from the assumed distribution (K-S; p<0.001). Table 1 summarizes the basic demographic data and trait-based measures for the study group. Figure 1 illustrates the distribution of age and the psychological assessments.

**Table 1:** Demographic data of the study population and summary of psychological evaluations.

	Total population	Females	Males
n	127	49	78
Age	35.8±12.8 (18–65)	36.3±13.2 (19–64)	35.5±12.6 (18–65)
Full Scale IQ	108.3±13.1 (74–137)	107.9±13.4 (80–137)	108.5±13.1 (74–136)
Verbal IQ	106.9±13 (67–134)	106.4±12.9 (79–129)	107.1±13.2 (67–134)
ASSQ Score	5.2±7.9 (0–39)	4±5.9 (0–22)	6±8.9 (0–39)
SRS Total Score	27.2±19.4 (0–109)	22±14.6 (1–71)	30.4±21.4 (0–109)

Data are given in mean ± standard deviation (range: minimum – maximum) format. ASSQ: Autism Spectrum Screening Questionnaire, SRS: Social Responsiveness Scale.

### Imaging protocols

DTI and fMRI scans were acquired on a 3.0 T MRI system (Magnetom Trio Tim, Siemens, Erlangen, Germany) using a spin echo echo-planar imaging (EPI) sequence (TR = 10000 ms, TE = 91 ms) with the GRAPPA parallel imaging technique (acceleration factor: 3). Diffusion-weighting gradients were applied in 64 different directions, b-value: 1000 s/mm^2^. Volumes consisted of 58 transverse slices, slice thickness: 2 mm, voxel size: 2 mm * 2 mm, matrix size: 128 * 128 (field of view: 256 mm). Resting-state functional magnetic resonance imaging protocol was adapted from the Brain Genomics Superstruct Project common acquisition protocol, provided courtesy of Randy Buckner. Over an experimental period of approximately 10 minutes, 260 volumes were acquired with a spin echo EPI sequence (TR = 2500 ms, TE = 30 ms). 38 axial slices were acquired with a matrix resolution of 72 * 72 (field of view: 216 mm), isotropic voxel size was 3 mm.

### Image processing – spatial standardization

Diffusion tensor and fMRI data were standardized to the MNI152 neuroimaging space. For guiding the transformation of the DTI space into the standard space, the fractional anisotropy (FA) images were used. Initially, FA images were registered to the FMRIB58 fractional anisotropy template linearly (FLIRT algorithm, 12 degrees of freedom registration using normalized correlation cost function, included in the FSL software library [41]). This affine transformation served as an initial stage for the non-linear registration (FNIRT algorithm).

The rs-fMRI data of each participant were realigned to the middle scan of the series using rigid body registration (MCFLIRT algorithm in FSL) [42]–[44] to adjust for motion and then co-registered to the T1-weighted MPRAGE image using rigid-body algorithm (FLIRT, FSL) [45]–[47]. Data were then spatially standardized by applying the warp field produced by the nonlinear co-registration of the skull-stripped [48] MPRAGE scan to the MNI152 non-linear template [49], [50] using the FNIRT tool in FSL. The diffusion-weighted and T1-weighted scans were skull-stripped using the BET command in FSL.

### Image processing – brain region system

A customized brain region system was defined to comprise the entire cortex and subcortical areas including the cerebellum. When determining the nodes of the network, Smith et al. showed that it is of crucial importance to ensure that the ROIs provide whole gray matter coverage and thus eliminate the deleterious effect of shared extra-network inputs [51].

For this purpose, we constructed a custom region system containing 149 regions, based on multiple brain atlases. Atlas data were available in MNI152 standard space. Regions were sampled from the Harvard-Oxford Cortical and Subcortical Structural [52]–[55] atlas while cerebellar labels were taken from the UCL (University College, London) probabilistic cerebellar atlas [56]. The thalamus region of the Harvard-Oxford atlas was parceled into seven sub-regions based upon the Oxford Thalamic Connectivity Atlas [57], [58], each subdivision representing thalamic connectivity domains to major cortical areas. Each atlas comprised voxel-wise probabilities of the volumetric label maps. Accessing this data, we delineated each ROI at p>0.25 probability threshold, using the in-house developed BrainLOC (Software Access: www.minipetct.hu/brainloc) software package. To eliminate regional overlaps, voxels belonging simultaneously to two or more ROIs were assigned to the one with the highest probability. We provide a complete list of label names, abbreviations and data sources in Table S1.

### Image processing - diffusion tensor estimation

Before the non-linear standardization of DTI acquisitions, tensors were fitted to the observed DWI signal using the DTIFIT algorithm (FSL), this step included the calculation of fractional anisotropy (FA) values. For the equation describing the relationship between the tensorial eigenvalues and FA, we refer to the commonly used ways in the literature [59]. Atlas-based labels were used to extract the regional values of FA. To determine the mean anisotropy value for each region, anisotropy measurements in each image voxel were weighted with the corresponding atlas-based probabilities.

### Image processing and network construction - resting state fMRI

We characterized the strength of temporal coherence (functional connectivity) between each pair of brain regions with the wavelet correlations of the averaged regional BOLD signals [60] on a low (<0.1 Hz) frequency band, which is most commonly used in rs-fMRI studies [61], [62]. For estimating wavelet correlations we applied four level maximum overlap discrete wavelet transformation [63], [64] with a Daubechies least asymmetric wavelets of length 8 (so called LA(8) wavelet) to calculate four sets of wavelet coefficients for each BOLD signal. The four level wavelet decomposition refers to four frequency bands (0.1–0.2 Hz, 0.05–0.1 Hz, 0.025–0.05 Hz, 0.0125–0.025 Hz) depending on the [2^1/(k+1)^/TR – 2^1/k^/TR, k = 1…4] rule and the TR = 2500 ms repetition time, we chose the coefficients related to the 0.025–0.05 Hz frequency range for the further analysis (Band 3). The wavelet correlation for this band was calculated from the wavelet coefficients as described in [63], [64].

**Figure 2:**
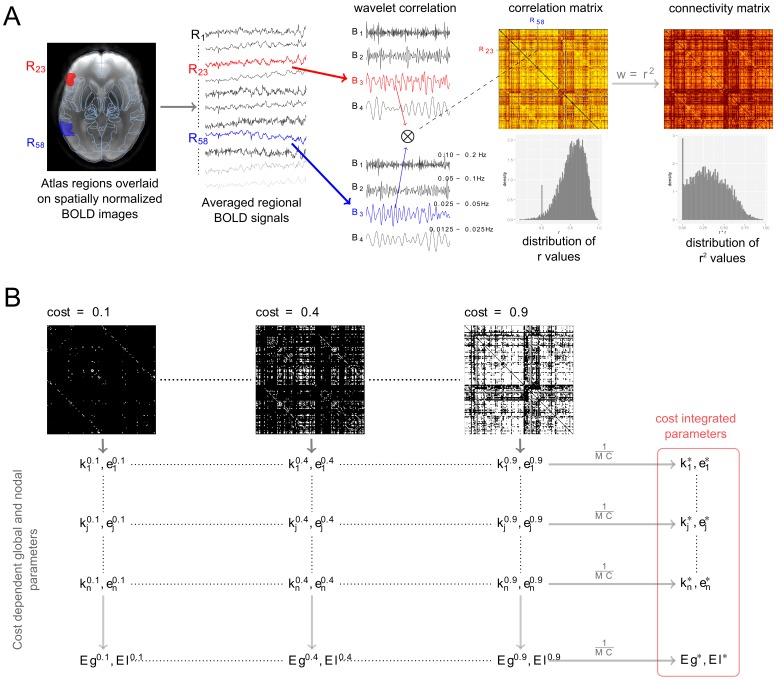
Processing pipeline of resting state functional MRI data. Panel A, far left: two atlas regions, Inferior Frontal Gyrus, triangular part (red, R23) and Middle Temporal Gyrus temporooccipital part (blue, R58) were overlaid on an atlas-space aligned fMRI image. The corresponding regional BOLD curves and their wavelet coefficients are shown on next images. Accordingly, the TR = 2500 ms repetition time and the four level wavelet decomposition the evaluated wavelet coefficients of four frequency bands (0.1–0.2 Hz, 0.05–0.1 Hz, 0.025–0.05 Hz, 0.0125–0.025 Hz) were generated (middle bottom image). In the 4th column the evaluated wavelet correlation matrix (top) and the distribution of correlation coefficients (bottom) are shown. We used these wavelet correlation values between each pair of brain regions to construct the matrix in which yellow color represents high correlation coefficients while red means low values. Panel B: In the bottom row, three adjacency matrices are shown with wired costs 0.1, 0.4 and 0.9. These matrices were generated from the weighted connectivity matrix (4^th^ panel, right bottom image) by different weight thresholds. Vertical gray arrows represent the calculation procedure of nodal and global network parameters at different cost levels. The horizontal gray arrows demonstrate the final step of Monte-Carlo based cost-integration procedure in which the summed parameters are divided by the integration steps (MC).

Wavelet correlations (r_ij_) between each possible pair of brain regions were computed and stored in 149*149 symmetric correlation matrices. In order to preserve the continuous nature of the correlation information we chose the weighted undirected network model for characterizing network properties [65]–[67]. To emphasize strong correlations and punish weak correlations we defined the connectivity between two regions as a power 2 of the absolute value of the wavelet correlation coefficients [68]: w_ij_ = r_ij_^2^. Using this soft threshold approach we produced 149*149 weighted connectivity matrices for each subject. Figure 2 summarizes the image processing pipeline that was utilized for the rs-fMRI data.

### Graph theoretical analysis of functional connectivities

In graph theory, the mathematical abstraction of a network is a *G* graph containing a set of N nodes and M edges. This graph can be represented as an N*N square “A” adjacency matrix with elements a_ij_ = 1 or 0, depending on whether an edge does (1) or does not exists (0) between nodes i and j. In the case of weighted networks the W connectivity/weight matrix containing w_ij_ elements represents the strength of connections between nodes i and j. In MRI-based functional brain network analysis the adjacency and weight matrices are *zero-diagonal, symmetric matrices* (w_ij_ = a_ij_ = 0, w_ij_ = w_ji_, a_ij_ = a_ji_) since modeling regional self-connections (i = j) and directed regional connections (w_ij_≠w_ji_, a_ij_≠a_ji_) are meaningless. These matrix properties correspond to *undirected binary* (so called unweighted) or *undirected weighted* graphs, depending on the used edge model (binary, weighted). In the case of weighted functional brain network without losing generality we assume that the weights of edges (i.e. the strength of connections) lie in the unit interval, [0,1].

### Measurement of graph sparsity

The number of maximum edges of an undirected graph M_C_ = N(N−1)/2, i.e. the number of elements of the upper-triangle of adjacency or weight matrix. The C index stands for “complete graph” in which every pair of nodes are connected. In the case of binary undirected graphs the
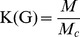
(Eq. 1)ratio defines the *edge density* or *wiring cost of G*. It is obvious that this parameter lies in the unit interval, [0,1], while the number of edges of empty and complete graphs is 0 and M_C_, respectively.

Substitution M and M_C_ into formula of K(G):
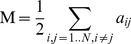
(Eq. 2)and so
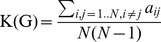
(Eq. 3)results in the fact that the wiring cost is the mean of values of the off-diagonal elements of the adjacency matrix. The adaptation of wiring cost definition of binary graphs to weighted graphs is simple [69], because the K(G) can be calculated as the mean value of the off-diagonal elements of weight matrix, if the weight values lie in the unit interval, with maximal value 1:
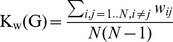
(Eq. 4)


### Network topology: global graph features

Using the described graph theoretical notation we can formulate a large set of parameters that characterize the global, nodal or modular properties of the investigated networks, as reviewed by Rubinov and Sporns [67]. It is known from the literature that the functional human brain network shows small-world characteristics, therefore in our study we focused on quantifying only those nodal and global parameters that may have an influence on this property. Latora and Marchiori [70] introduced the efficiency-based characterization of small-world networks, which, due to its computational benefits, was proved to be more effective than the classical *characteristic path length* and *clustering coefficient* based calculation methods [71], [72]. The *efficiency* was introduced as the measure of the effectiveness of information exchange between nodes, while the average efficiency of nodes of a *G* graph was defined as *global efficiency (Eg)*, which gives a normalized measure (lies in [0,1]) of the information transfer efficiency of parallel systems. The normalized *local efficiency (El)* of the network measures how efficient the local communication between first neighbors of a node is if this node is faulted or removed (fault tolerance). Latora and Marchiori also showed that the small-world behavioral network has high global and local efficiency [70]. According to this assumption, we calculated two nodal (degree and efficiency) and two global (global and local efficiency) parameters for all subjects networks using the following formulas.

### Nodal parameters of unweighted graphs

The *k_i_ node degree* [67] is defined as the sum of edges of node i:
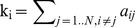
(Eq. 5)The efficiency coefficient of the path (path efficiencies) between node *i* and *j* is defined as the inverse of the *l_ij_ shortest distance* between these nodes [67]. The shortest path length *l_ij_* between nodes *i* and *j* means the minimum of all possible path lengths between nodes *i* and *j*, and can be calculated from the adjacency matrix by Dijksra's [73] or Floyd's [74] algorithms. Using these definitions the *efficiency of node i* is defined as the average of the path efficiencies of the given node:
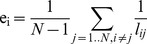
(Eq. 6)


### Global graph parameters of unweighted graphs

Global efficiency E_g_ is given as the mean of nodal efficiencies and gives a normalized measure (lies in [0,1]) of the information transfer efficiency of the network:
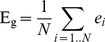
(Eq. 7)Local efficiency of an unweighted graph is defined as the average global efficiency of sub-graphs of nodes, because it measures how efficient the local communication is between the first neighbors of a node if this node is faulted or removed:
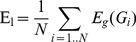
(Eq. 8)Where G_i_ denotes the subgraph composed from the neighbors of node i, and by definition does not contain node i.

### Cost-integrated measurement of topological metrics

One of the critical points of functional brain network analysis is the connection (edge) selection or the thresholding procedure. The fundamental aim of this process is to eliminate weak connections from the graph representation of network by an arbitrarily chosen weight threshold. The result can be a weighted graph containing only edges with higher weight than the applied threshold or it can be an unweighted graph containing only thresholded edges. Thresholding will change the wiring cost of graphs, in other words, it changes the topology of the investigated network. In the functional brain network assays we have an illusory solution to avoid this problem if we use the evaluated, unthresholded primary networks for any population level analysis. However, the primary networks differ in their average correlation coefficients and they also differ in their mean weights and wired-costs [75], which makes difficult to compare their topology parameters. Although, the adaptive thresholding technique [76] could guarantee the same weighted or unweighted wired cost for all networks in the population by uniquely selected thresholds, but it generates new questions: which wired cost is the best for the analysis and how the selected cost affects the results? Since there is currently no consensus in the literature regarding selection of threshold for weighted graph analysis we applied a cost-integrated technique as introduced by Ginestet *et.al.* in [69]. Following the definition of this paper we calculate the cost-integrated values of any X topological parameter of the graph *G* by this formula:
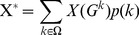
(Eq. 9)where k is a cost value from the Ω set (set of all possible cost values), and p(k) the denotes the probability of the occurrence of k cost. The *G* ^k^ is an *unweighted graph* having k wired cost and it is generated from *G by an appropriate threshold* and X(*G* ^k^) is the value of the X topological parameter calculated on *G* ^k^. We assumed that k has uniform distribution, which means that the p(k) = 1/M_c_, so the cost-integrated version of any X can be calculated as follows:
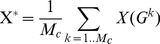
(Eq. 10)In our case, the number of nodes is N = 149 and so the M_C_ = 11026, we had to apply the Monte-Carlo (MC) based estimation to calculate cost integrated values (described also in [69]). Our preliminary analysis showed that in the case of 5 randomly selected subjects 200 MC-sample gave correct E_g_ and E_l_ values, so this sample rate was used in this study. The cost integration range was set as [0.05, 0.95].

In our special case the cost-integrated nodal degree (k_i_*), nodal efficiency (e_i_*), global efficiency (Eg*) and local efficiency (El*) were calculated for a subject as described in the Document S1 and illustrated in Panel B of Figure 2.

### Software environment

For the network generation and for the cost-integrated evaluation of topological metrics we used FSL and the following in-house developed software:

**BrainLOC** (www.minipetct.com/brainloc) atlas database management software was used to generate 149-regional database from different atlases end evaluate averaged regional BOLD curves.

**BrainNetTools** parallelized utilities running on the high-performance cluster (HPC) of University of Debrecen were used to generate wavelet correlation matrices, connectivity matrices and to calculate cost-integrated topological metrics.

**BrainCON** (www.minipetct.com/braincon) was used for network visualization.

The components of BrainNetTool were validated by the following external software.

- Brain Connectivity Toolbox (BCT, http://www.brain-connectivity-toolbox.net/), release 29/03/2012.
- igraph 0.6 R-package (http://igraph.sourceforge.net/)
- wavelets 0.2–7 R-package (http://cran.r-project.org/web/packages/wavelets/index.html)
- NetworkAnalysis 0.3–1 R-package (http://cran.r-project.org/package=NetworkAnalysis)

### Utilizing the statistical parametric network approach

The statistical parametric network (SPN) terminology was introduced by Ginestet and Simmons [77]. We used the population level mean SPN to demonstrate the subgraph of regional connectivity system. The generated mean SPN provides a method to statistically infer the mean wavelet correlation matrix of the population, and it contains z-scores of correlation coefficients:
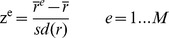
(Eq. 11)Where r^−^ and sd(r) stand for the grand sample mean and grand sample standard deviation. The r^e^

denotes the mean correlation coefficients of the edge e:
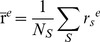
(Eq. 12)

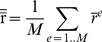
(Eq. 13)


### Correlation model, covariates and contrasts

A general linear model (GLM) based variance analysis was performed. The effects of the dimensional autistic traits (ASSQ and SRS scores) on the relevant graph theoretical and diffusion anisotropy were tested, age and gender terms were added as covariates. Random effects were modeled by using the randomly assigned subject identifier in the model. The general model was formulated according to:
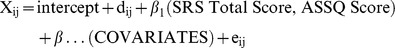
where X_ij_ is the i^th^ graph-theory based global or local topological descriptor or the regional diffusion anisotropy for the j^th^ subject. Intercept and β terms are fixed effects (β_1_: explanatory variables – psychological scores, covariates: age, gender or scanner dependency), d_ij_ stands for random effects (within-person dependence) and e_ij_ is the residual error. In our study design, we used two different sets of covariates. Here we describe these models by listing the relevant dependent variables or explanatory variables, factors and covariates. The hypothesized interaction is defined by a contrast matrix in which the columns correspond to the elements of the parameter vector.

The first model assumes an inverse relationship between the psychological measurements and the predicted variable (i.e. functional topology or diffusion anisotropy). Age, gender and subject identifier are taken as covariates. The relevant parameter vector and contrast matrix are described below.
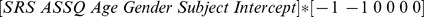
We hypothesize that the intelligence profile (i.e. performance IQ and verbal IQ) interacts with regional graph theoretical parameters and also correlates with the autistic traits. Therefore we aimed to utilize a model which is adjusted for this putative effect. The intelligence profile is commonly characterized by discrepant values of performance and verbal IQ scores [38] although the direct relationship between HFA and IQ discrepancy has been questioned [39]. In our study population, verbal IQ significantly and positively correlated with performance IQ (Pearson's r = 0.55; p<0.001) while negative correlation was discovered between the verbal IQ and the ASSQ score (r = −0.393; p<0.001) and the SRS score (r = −0.343; p<0.001). Hence in the second model, we controlled the effects of the WASI verbal IQ profile:
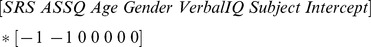
Custom scripts in IBM SPSS Statistics for Windows 20 were used to perform multiple univariate GLM tests for the 149 brain regions. The effects of the autistic trait were tested with variance statistics, for each test we provide the F values and uncorrected p-values. The contribution of each variable to the overall variance is characterized by reporting the slope (B) and the relevant significance values. The effect size is estimated by the partial eta squared value, which is calculated using the following equation:
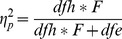
(Eq. 14)where *F* is the test statistic and *dfh* and *dfe* are its degrees of freedom and degrees of freedom for error. For the interpretation of effect size, we refer to the rule of thumb by Cohen, where similarly to r^2^ and R^2^ values, 0.01 denotes small, 0.06 moderate and >0.14 marks large correlation [78]. However, it is also important to note that partial eta measures tend to overestimate the effect size.

The relevant software syntax is described in the Document S2.

### False discovery rate correction

Due to the exploratory design of our study and the large number of brain regions evaluated, it was necessary to account for multiple comparisons during hypothesis testing. Here we report results as if the 149 measurements (i.e. in each evaluated brain region) were independent. After obtaining the p-value vectors for the network degree, efficiency and regional white matter anisostropy, we utilized the Benjamini-Hochberg (BH) procedure for false discovery rate (FDR) control [79], with α level set at 0.05. The relevant SPSS syntax of the correction procedure is described in the Document S2.

## Results

### Validation of network measurements

**Figure 3:**
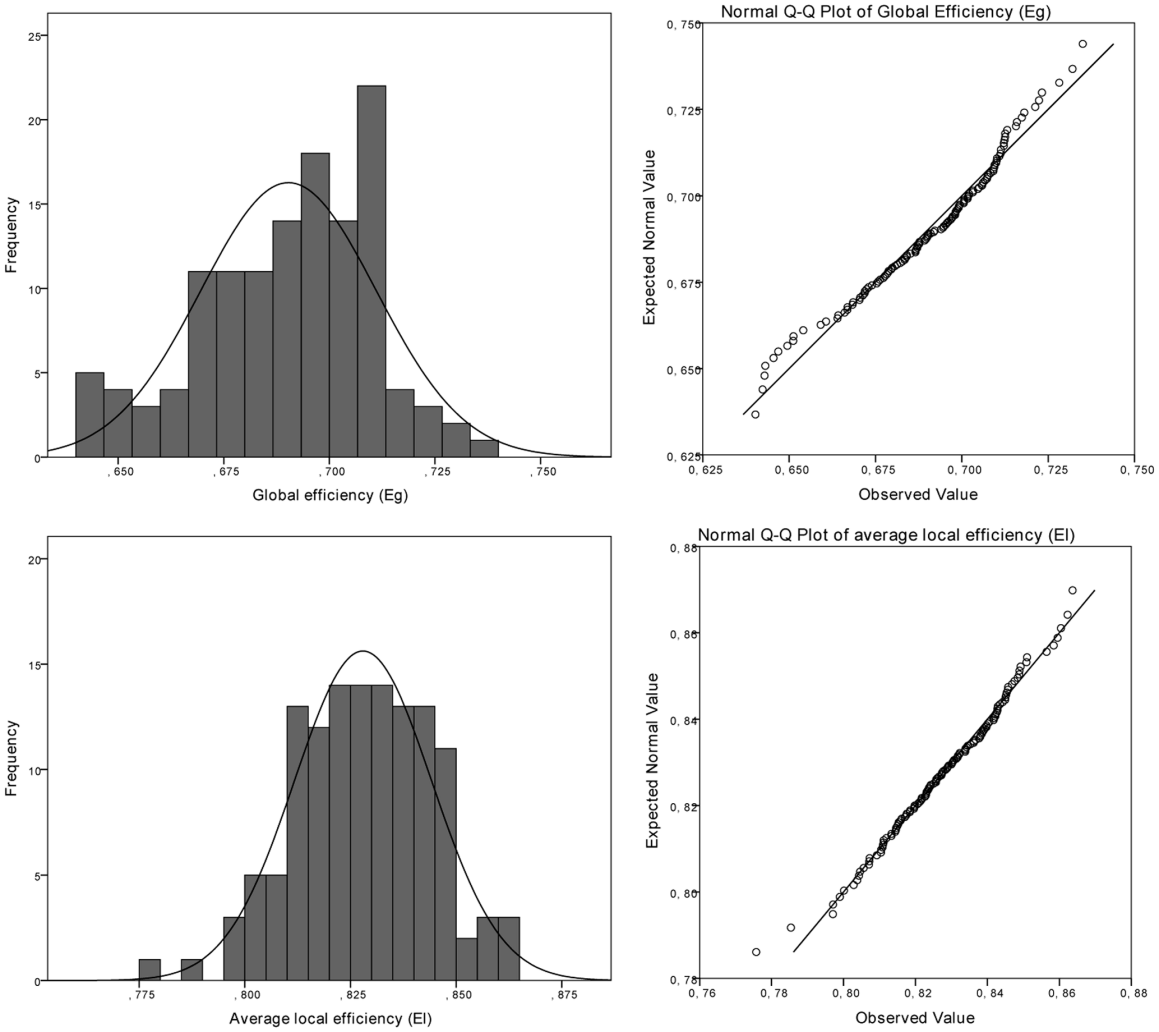
Distribution of global functional network properties. Global efficiency (E_g_) and average local efficiency (E_l_). Data are depicted as histograms (left panels) and Q-Q (quantile-quantile) probability plots (right panels) in which reference lines of the normal distribution are given (n = 127).

**Figure 4:**
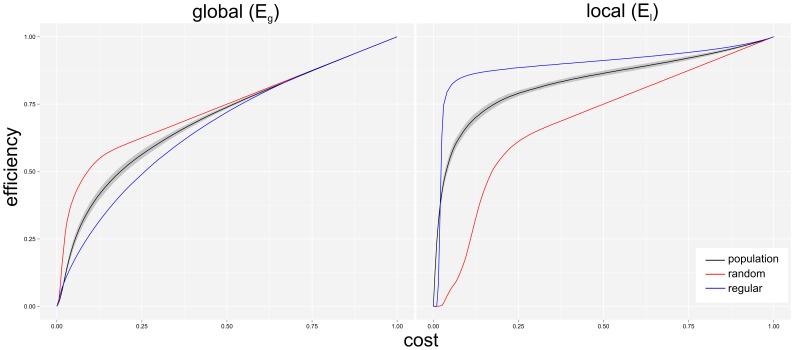
Small-world properties of functional and synthetic networks. Global (E_g_) and local (E_l_) efficiencies are depicted as a function of wired-cost for random (red), a regular (blue) and the investigated human brain networks. In latter case, the averages (black line) and the standard deviation (gray band) of efficiency values are shown. The E_g_ and E_l_ of brain networks monotonically increase by the cost with relatively low standard deviation. This means that all brain networks have simultaneously high E_g_ and E_l_ values in the [0.34–0.5] cost-range, which verify that these networks have small-world properties.

We analyzed the population level cost-dependent distribution of E_g_ and E_l_ parameters, which is illustrated in Figure 3. Figure 4 demonstrates that the E_g_ and E_l_ of the brain networks monotonically increase by the cost with relatively low standard deviation. It means that all brain networks have high E_g_ and E_l_ values in the [0.34–0.5] cost-range, a conservatively defined small-world regime [80], which verifies that these networks have small-world properties [70]. We also calculated the cost-dependent efficiencies for random (red) and regular (blue) networks. In the case of random network containing 149 nodes, similarly to the brain networks, the edges were randomly generated in proportion to the given wired cost. The calculation was repeated 30 times for evaluating averaged cost-dependent E_g_ and E_l_ values. Efficiencies of regular networks were also calculated from 149-node networks, but in this case the required edge density was guaranteed by regularly distributed edges. It seems that the brain networks are better suited for efficiency-based small-world criteria (*high E_g_ and high E_l_*) than the regular and random network networks, i.e. at any cost level; the brain networks have higher E_g_ than the regular and higher E_l_ values than the random networks.

### Global functional connectivity

Global brain functional connectivity was portrayed by two graph theoretical parameters (E_g_, E_l_); here we describe their observed characteristics and interactions with the phenotypic descriptors. All parameters were normally distributed in the study cohort (K-S test; asymptotic significances; E_g_: p = 0.63; E_l_: p = 0.99). The factor gender was not associated with any of the global graph theoretical parameters, age showed significant positive correlation with E_l_ (η_p_^2^: 0.046; F = 6.54; p = 0.012). WASI verbal IQ scores did not correlate with the connectivity parameters (controlling for age and gender). ASSQ values were found to significantly predict the averaged local efficiency parameter E_l_ when controlling for the effects of age and gender (η_p_^2^ = 0.046; F = 6.33; p = 0.013); the SRS Total Score alone did not show such effect. Similarly to the analysis of regional parameters, a combined model of the ASSQ and SRS, controlling for age and gender effects was utilized. This revealed small, significant association between the psychological trait and average local efficiency E_l_ (η_p_^2^ = 0.047; F = 5.99; p = 0.016). When controlling the model for the effects of the verbal IQ profile, the correlation with average local efficiency was slightly reduced (η_p_^2^ = 0.038; F = 4.39; p = 0.038). Numeric data of the described interactions are given in Table 2.

**Table 2:** Global graph theoretical network correlates of autistic traits, age, gender and IQ using GLM.

Dependent variables in the GLM design	Covariates in GLM (variables to control)	Association with Global efficiency E_g_	Association with Average local efficiency E_l_
Age	-	F: 0.25	F: 6.54
		p: 0.62	p: 0.012*
		η_p_^2^: 0.002	η_p_^2^: 0.046
		B_age_: 6.07*10^−5^	B_age_: 2.59*10^−4^
Gender	-	F: 0.55	F: 2.02
		p: 0.46	p: 0.16
		η_p_^2^: 0.004	η_p_^2^: 0.015
		B_Gender_: 2.67*10^−3^	B_Gender_: 3.9*10^−3^
WASI Verbal IQ	Age, Gender	**F: 1.97**	**F: 0.68**
		**p: 0.16**	**p: 0.41**
		**η_p_^2^: 0.016**	**η_p_^2^: 0.0055**
		B_WASI_: 1.94*10^−4^ (η_p_^2^: 0.016; p: 0.16)	B_WASI_: 8.79*10^−5^ (η_p_^2^: 0.0055; p: 0.41)
		B_Age_: 4.59*10^−5^ (η_p_^2^: 8.6*10^−4^; p: 0.75)	B_Age_: 2.45*10^−4^ (η_p_^2^: 0.04; p: 0.026)*
		B_Gender_: 8.8*10^−4^ (η_p_^2^: 4.57*10^−4^; p: 0.81)	B_Gender_: 0.0035 (η_p_^2^: 0.012; p: 0.22)
ASSQ Score	Age, Gender	**F: 0.051**	**F: 6.33**
		**p: 0.82**	**p: 0.013** *
		**η_p_^2^: 3.8** * **10^−4^**	**η_p_^2^: 0.046**
		B_ASSQ_: 5.24*10^−5^ (η_p_^2^: 3.8*10^−4^; p: 0.82)	B_ASSQ_: −4.28*10^−4^ (η_p_^2^: 0.046; p: 0.013)*
		B_Age_: 6.21*10^−5^ (η_p_^2^: 0.0016; p: 0.65)	B_Age_: 2.45*10^−3^ (η_p_^2^: 0.044; p: 0.015)*
		B_Gender_: 0.003 (η_p_^2^: 0.0037; p: 0.49)	B_Gender_: 0.0048 (η_p_^2^: 0.024; p: 0.075)
SRS Total Score	Age, Gender	**F: 1.09**	**F: 1.36**
		**p: 0.29**	**p: 0.25**
		**η_p_^2^: 0.0088**	**η_p_^2^: 0.011**
		B_SRS_: −1.04*10^−4^ (η_p_^2^: 0.0088; p: 0.29)	B_SRS_: −8.72*10^−5^ (η_p_^2^: 0.011; p: 0.25)
		B_Age_: 4.92*10^−5^ (η_p_^2^: 9.2*10^−3^; p: 0.74)	B_Age_: 2.56*10^−4^ (η_p_^2^: 0.042; p: 0.022)*
		B_Gender_: 0.0046 (η_p_^2^: 0.011; p: 0.25)	B_Gender_: 0.005 (η_p_^2^: 0.023; p: 0.091)
ASSQ*SRS Total Score	Age, Gender	**F: 0.025**	**F: 5.99**
		**p: 0.88**	**p: 0.016** *
		**η_p_^2^: 2.06** * **10^−3^**	**η_p_^2^: 0.047**
		B_SRS_: −1.23*10^−4^ (η_p_^2^: 8.6*10^−3^; p: 0.31)	B_SRS_: 1.95*10^−5^ (η_p_^2^: 4*10^−4^; p: 0.83)
		B_ASSQ_: 8.46*10^−5^ (η_p_^2^: 7.02*10^−3^; p: 0.77)	B_ASSQ_: −4.62*10^−4^ (η_p_^2^: 0.037; p: 0.033)*
		B_Age_: 5.65*10^−5^ (η_p_^2^: 1.2*10^−3^; p: 0.71)	B_Age_: 2.16*10^−4^ (η_p_^2^: 0.031; p: 0.053)
		B_Gender_: 4.58*10^−3^ (η_p_^2^: 0.011 p: 0.25)	B_Gender_: 0.0049 (η_p_^2^: 0.024; p: 0.089)
ASSQ*SRS Total Score	Age, Gender, WASI Verbal IQ	**F: 0.77**	**F: 4.39**
		**p: 0.39**	**p: 0.038** *
		**η_p_^2^: 0.007**	**η_p_^2^: 0.038**
		B_SRS_: −9.14*10^−5^ (η_p_^2^: 0.0047; p: 0.47)	B_SRS_: 5.14*10^−6^ (η_p_^2^: 2.5*10^−5^; p: 0.96)
		B_ASSQ_: 3.2*10^−4^ (η_p_^2^: 0.01; p: 0.28)	B_ASSQ_: −4.26*10^−4^ (η_p_^2^: 0.03; p: 0.065)
		B_Age_: 3.73*10^−5^ (η_p_^2^: 5.2*10^−3^; p: 0.81)	B_Age_: 1.88*10^−4^ (η_p_^2^: 0.022; p: 0.12)
		B_VIQ_: 3*10^−3^ (η_p_^2^: 0.028; p: 0.078)	B_VIQ_: −7.34 *10^−6^ (η_p_^2^: 2.9*10^−5^; p: 0.96)
		B_Gender_: 0.0023 (η_p_^2^: 0.0028; p: 0.58)	B_Gender_: 4.8*10^−3^ (η_p_^2^: 0.021 p: 0.13)

GLM: General Linear Model, ASSQ: Autism Spectrum Screening Questionnaire, SRS: Social Responsiveness Scale.

* : p<0.05

### Regional functional connectivities and diffusion anisotropy

The regional degree and efficiency values were predominantly normally distributed in the study population (K-S tests). In the first part of the regional evaluations, we tested the joint effects of the ASSQ and SRS on graph theoretical parameters. This model was adjusted to control for the subject's age and gender. Significant inverse, moderate correlation was discovered between the autistic traits and the network degree and efficiency of the left and right posterior cingulate gyrus, although after FDR adjustment, only that of the right posterior cingulate gyrus remained statistically significant (nodal degree: η_p_^2^ = 0.11; F = 14.98; p = 0.00018; nodal efficiency: η_p_^2^ = 0.11; F = 14.43; p = 0.00024). The diffusion anisotropy of these two areas did not present significant association with the examined autistic traits. After accounting for contributions of age, gender and random effects, we found that higher scores on the SRS and ASSQ – indicating greater autistic traits – were associated with lower diffusion anisotropy in the right parahippocampal gyrus, posterior part (η_p_^2^ = 0.069; F = 8.81; p = 0.0036) and right temporal fusiform cortex, posterior part (η_p_^2^ = 0.054; F = 6.82; p = 0.01). However, we emphasize that this interaction was not proven to be significant after FDR adjustment by the BH procedure. We summarized the results of this analysis in Table 3.

**Table 3:** Regional neural correlates of self-reported autistic traits, GLM design 1.

Region name	Nodal Degree	Nodal Efficiency	Regional Diffusion Anisotropy
Right cingulate gyrus, posterior part	**F: 14.98**	**F: 14.34**	**F: 0.27**
	**p: 0.00018** *** †	**p: 0.00024** *** †	**p: 0.61**
	**η_p_^2^: 0.11**	**η_p_^2^: 0.11**	**η_p_^2^: 0.002**
	B_ASSQ_: −0.51 (η_p_^2^: 0.11; p: 1.64*10^−4^)***	B_ASSQ_: −1.9*10^−3^ (η_p_^2^: 0.11; p: 1.8*10^−4^)**	B_ASSQ_: −7.15*10^−5^ (η_p_^2^: 0.002; p: 0.65)
	B_SRS_: 0.083 (η_p_^2^: 0.019; p: 0.13)	B_SRS_: 3.5*10^−4^ (η_p_^2^: 0.022; p: 0.1)	B_SRS_: 2.37*10^−6^ (η_p_^2^: 1.1*10^−5^; p: 0.97)
	B_Age_: −0.018 (η_p_^2^: 0.001; p: 0.79)	B_Age_: −8.8*10^−5^ (η_p_^2^: 0.001; p: 0.74)	B_Age_: −9.47*10^−7^ (η_p_^2^: 0.012; p: 0.23)
	B_Gender_: −0.41 (η_p_^2^: 4.33*10^−4^; p: 0.82)	B_Gender_: −1.8*10^−3^ (η_p_^2^: 5.8*10^−4^; p: 0.79)	B_Gender_: 4*10^−3^ (η_p_^2^: 0.034; p: 0.044)*
Left cingulate gyrus, posterior part	**F: 8.05**	**F: 7.18**	**F: 0.21**
	**p: 0.0053** **	**p: 0.0084** **	**p: 0.65**
	**η_p_^2^: 0.062**	**η_p_^2^: 0.056**	**η_p_^2^: 0.002**
	B_ASSQ_: −0.37 (η_p_^2^: 0.068; p: 0.004)**	B_ASSQ_: −1.4*10^−3^ (η_p_^2^: 0.065; p: 0.004)**	B_ASSQ_: 3.15*10^−5^ (η_p_^2^: 2.61*10^−4^; p: 0.86)
	B_SRS_: 0.072 (η_p_^2^: 0.016; p: 0.16)	B_SRS_: 3.08*10^−4^ (η_p_^2^: 0.02; p: 0.12)	B_SRS_: 3.78*10^−5^ (η_p_^2^: 0.002; p: 0.61)
	B_Age_: 0.062 (η_p_^2^: 0.008; p: 0.33)	B_Age_: 2.3*10^−4^ (η_p_^2^: 0.007; p: 0.34)	B_Age_: −1.11*10^−4^ (η_p_^2^: 0.012; p: 0.23)
	B_Gender_: −0.27 (η_p_^2^: 2.1*10^−4^; p: 0.88)	B_Gender_: −1.2*10^−3^ (η_p_^2^: 2.9*10^−4^; p: 0.85)	B_Gender_: 2.2*10^−3^ (η_p_^2^: 0.007; p: 0.36)
Right parahippocampal gyrus, posterior part	**F: 0.3**	**F: 0.34**	**F: 8.81**
	**p: 0.58**	**p: 0.56**	**p: 0.0036** **
	**η_p_^2^: 0.002**	**η_p_^2^: 0.003**	**η_p_^2^: 0.069**
	B_ASSQ_: 0.12 (η_p_^2^: 0.002; p: 0.66)	B_ASSQ_: 6.7*10^−4^ (η_p_^2^: 1.4*10^−3^; p: 0.69)	B_ASSQ_: −1.1*10^−3^ (η_p_^2^: 0.066; p: 0.005)**
	B_SRS_: 0.007 (η_p_^2^: 2.8*10^−5^; p: 0.95)	B_SRS_: 1.4*10^−4^ (η_p_^2^: 3.5*10^−4^; p: 0.84)	B_SRS_: 1.5*10^−4^ (η_p_^2^: 0.008; p: 0.34)
	B_Age_: 0.085 (η_p_^2^: 0.003; p: 0.54)	B_Age_: 1.52*10^−6^ (η_p_^2^: ∼0 p: 0.99)	B_Age_: 5.32*10^−5^ (η_p_^2^: 0.001; p: 0.78)
	B_Gender_: 6.34 (η_p_^2^: 0.024; p: 0.089)	B_Gender_: 0.051 (η_p_^2^: 0.04; p: 0.026)*	B_Gender_: 3.8*10^−3^ (η_p_^2^: 0.005; p: 0.45)
Right temporal fusiform cortex, posterior part	**F: 0.34**	**F: 0.51**	**F: 6.83**
	**p: 0.56**	**p: 0.48**	**p: 0.01** *
	**η_p_^2^: 0.003**	**η_p_^2^: 0.004**	**η_p_^2^: 0.054**
	B_ASSQ_: 0.14 (η_p_^2^: 0.003; p: 0.53)	B_ASSQ_: 7.5*10^−4^ (η_p_^2^: 0.004; p: 0.48)	B_ASSQ_: −6.7*10^−4^ (η_p_^2^: 0.051; p: 0.013)*
	B_SRS_: −0.029 (η_p_^2^: 0.001; p: 0.75)	B_SRS_: −1.11*10^−4^ (η_p_^2^: 0.001; p: 0.8)	B_SRS_: 8.95*10^−5^ (η_p_^2^: 0.005; p: 0.42)
	B_Age_: 0.084 (η_p_^2^: 0.005; p: 0.45)	B_Age_: 3.5*10^−4^ (η_p_^2^: 0.003; p: 0.53)	B_Age_: 9.46*10^−5^ (η_p_^2^: 0.004; p: 0.49)
	B_Gender_: 3.7 (η_p_^2^: 0.013; p: 0.21)	B_Gender_: 0.015 (η_p_^2^: 0.009; p: 0.29)	B_Gender_: −1.4*10^−3^ (η_p_^2^: 0.001; p: 0.71)

We demonstrate the joint effects of Social Responsiveness Scale (SRS Total Score) and Autism Spectrum Screening Questionnaire (ASSQ), controlling for age and gender. GLM: General Linear Model.

* : 0.01≤p<0.05.

** : 0.001≤p<0.01.

*** : p<0.001.

† : Significant interaction after false discovery rate correction with the Benjamini-Hochberg procedure.

**Figure 5:**
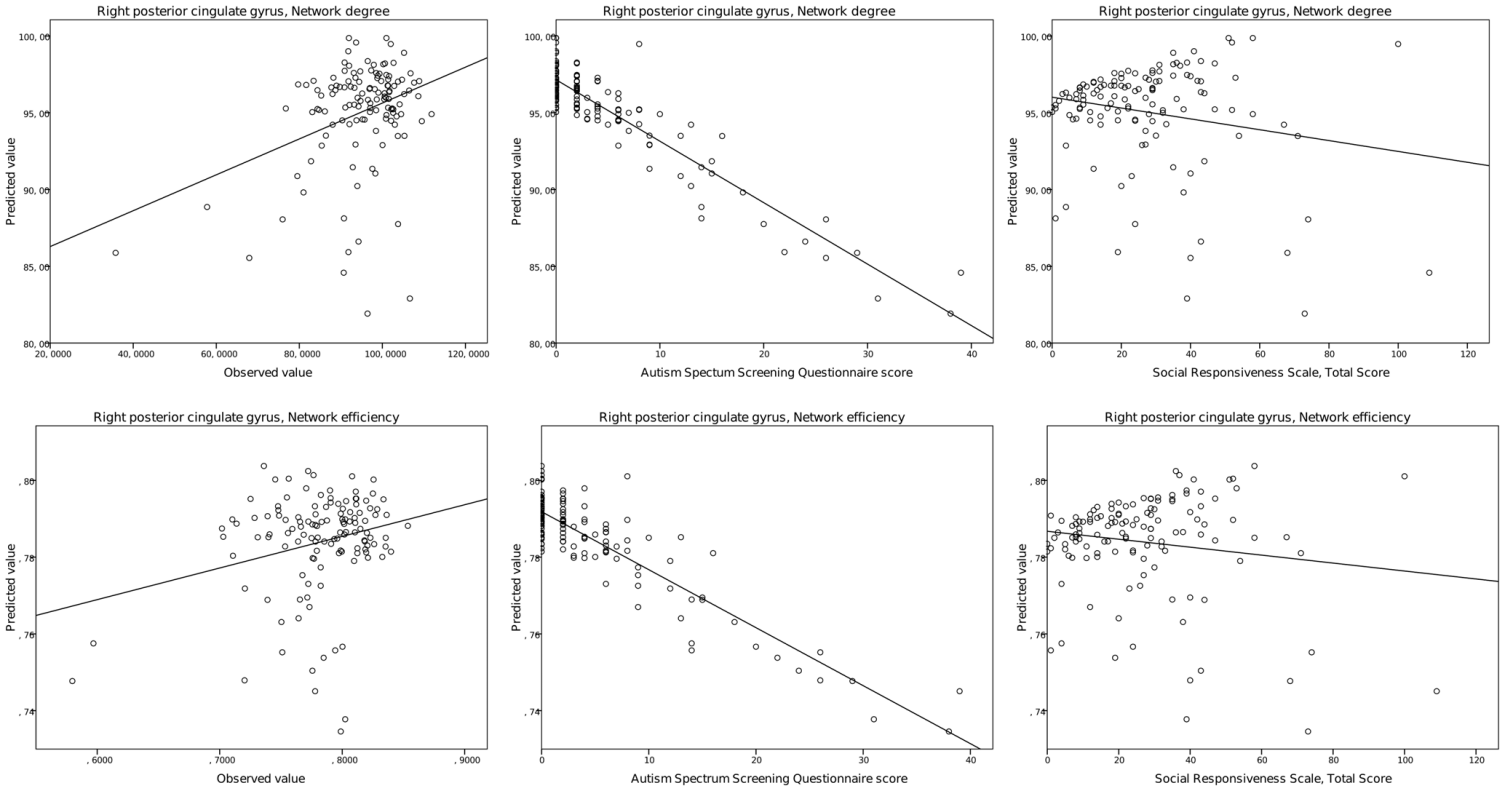
Regional graph theoretical correlates of self-reported autistic traits. Left panels: observed vs. predicted values of the dependent variables in the general linear model analysis. Middle panels: predicted values vs. ASSQ score. Right panels: predicted values vs. SRS total scores.

**Figure 6:**
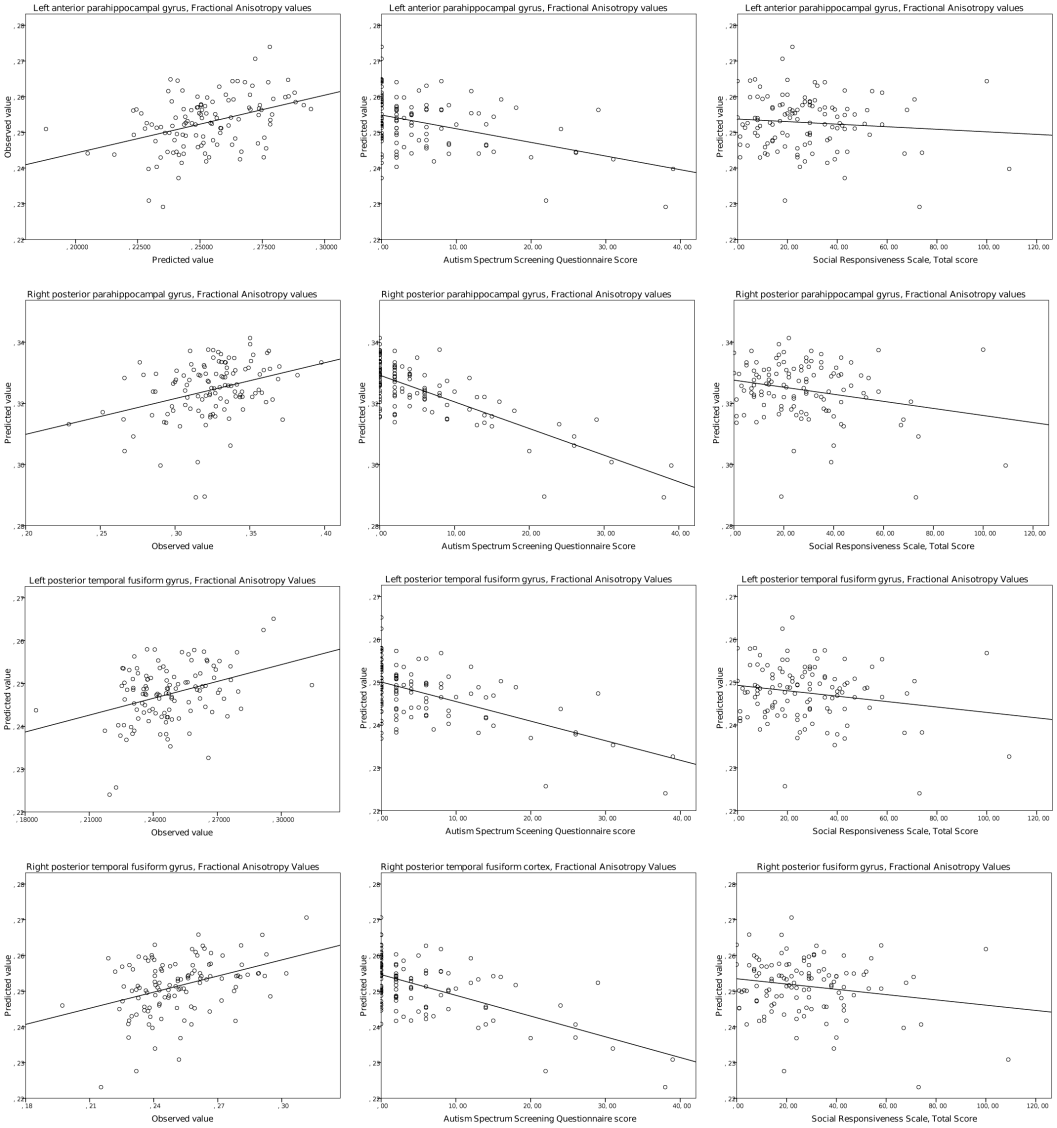
Regional diffusion anisotropy correlates of self-reported autistic traits. Left panels: observed vs. predicted values of the dependent variables in the general linear model analysis. Middle panels: predicted values vs. ASSQ score. Right panels: predicted values vs. SRS total scores.

In the next series of evaluations, we adjusted the GLM to account for the confounding effects of WASI verbal IQ. In this test, the putative interaction between regional graph theoretical variables and the verbal intelligence profile is controlled. The significance level of the interaction between autistic traits and right posterior cingulate gyrus degree and efficiency was mildly reduced but small correlations were found (degree: η_p_^2^ = 0.094; F = 11.46; p = 0.00098; efficiency: η_p_^2^ = 0.078; F = 9.45; p = 0.0026). After our conservative FDR adjustment procedure neither the left nor the right posterior cingulate gyrus showed significant association with the physiological markers, although the interaction effect in these regions still remained prominent when comparing to other areas. In this model, the diffusion anisotropy of the left anterior parahippocampal gyrus was found to be significantly correlating with the autistic trait (η_p_^2^ = 0.094; F = 11.46; p = 0.00098). Similar effect was noted for the right posterior parahippocampal gyrus (η_p_^2^ = 0.101; F = 12.29; p = 0.00066), left posterior temporal fusiform gyrus (η_p_^2^ = 0.093; F = 11.28; p = 0.0011) and the right posterior temporal fusiform gyrus (η_p_^2^ = 0.109; F = 13.46; p = 0.00038). The relationship of the predicted and observed values of graph measurements and diffusion anisotropy is depicted in Figure 5 and Figure 6. Supporting data are given in Table 4. Unadjusted p values for each brain region are listed in Table S2.

**Table 4:** Regional neural correlates of self-reported autistic traits, controlled for verbal IQ: GLM design 2.

Region name	Nodal Degree	Nodal Efficiency	Regional Diffusion Anisotropy
Right cingulate gyrus, posterior part	**F: 11.46**	**F: 9.45**	**F: 1.01**
	**p: 0.00098** ***	**p: 0.0026** **	**p: 0.32**
	**η_p_^2^: 0.094**	**η_p_^2^: 0.078**	**η_p_^2^: 0.0022**
	B_ASSQ_: −0.49 (η_p_^2^: 0.102; p: 5.6*10^−4^)***	B_ASSQ_: −1.8*10^−3^ (η_p_^2^: 0.092; p: 0.001)**	B_ASSQ_: −1.2*10^−4^ (η_p_^2^: 0.0047; p: 0.47)
	B_SRS_: 0.081 (η_p_^2^: 0.017; p: 0.17)	B_SRS_: 3.5*10^−4^ (η_p_^2^: 0.02; p: 0.13)	B_SRS_: −2.7*10^−5^ (η_p_^2^:1.3*10^−3^; p: 0.71)
	B_Age_: −0.017 (η_p_^2^: 4.7*10^−4^; p: 0.82)	B_Age_: −6.9*10^−5^ (η_p_^2^: 5.4*10^−4^; p: 0.81)	B_Age_: −7.6*10^−5^ (η_p_^2^: 0.0069; p: 0.38)
	B_viq_: 0.04 (η_p_^2^: 2.3*10^−3^; p: 0.61)	B_viq_: 2.9*10^−4^ (η_p_^2^: 7.9*10^−3^; p: 0.35)	B_viq_: −1.6*10^−4^ (η_p_^2^: 0.026; p: 0.088)
	B_Gender_: −0.25 (η_p_^2^: 1.6*10^−4^; p: 0.89)	B_Gender_: −1.9*10^−3^ (η_p_^2^: 6.2*10^−4^; p: 0.79)	B_Gender_: 0.0053(η_p_^2^: 0.049; p: 0.019)*
Left cingulate gyrus, posterior part	**F: 5.35**	**F: 3.82**	**F: 0.00**
	**p: 0.023** *	**p: 0.53**	**p: 1.00**
	**η_p_^2^: 0.046**	**η_p_^2^: 0.033**	**η_p_^2^: 0.0018**
	B_ASSQ_: −0.36 (η_p_^2^: 0.064; p: 0.007)**	B_ASSQ_: −1.3*10^−3^ (η_p_^2^: 0.053; p: 0.014)*	B_ASSQ_: −2.2*10^−5^ (η_p_^2^: 1.1*10^−4^; p: 0.91)
	B_SRS_: 0.095 (η_p_^2^: 0.025; p: 0.092)	B_SRS_: 3.9*10^−4^ (η_p_^2^: 0.03; p: 0.067)	B_SRS_: 1.4*10^−5^ (η_p_^2^: 2.8*10^−4^; p: 0.86)
	B_Age_: 0.074 (η_p_^2^: 0.01; p: 0.28)	B_Age_: 2.9*10^−4^ (η_p_^2^: 0.011; p: 0.28)	B_Age_: −1.5*10^−4^ (η_p_^2^: 0.021; p: 0.13)
	B_viq_: 0.071 (η_p_^2^: 0.008; p: 0.34)	B_viq_: 3.8*10^−4^ (η_p_^2^: 0.016; p: 0.19)	B_viq_: −1.3*10^−4^ (η_p_^2^: 0.014; p: 0.22)
	B_Gender_: −0.056 (η_p_^2^: 9*10^−6^; p: 0.97)	B_Gender_: −9.6*10^−4^ (η_p_^2^: 1.7*10^−4^; p: 0.89)	B_Gender_: 0.0028 (η_p_^2^: 0.01; p: 0.29)
Left parahippocampal gyrus, anterior part	**F: 0.37**	**F: 0.83**	**F: 11.46**
	**p: 0.544**	**p: 0.36**	**p: 0.00098** *** †
	**η_p_^2^: 0.003**	**η_p_^2^: 0.007**	**η_p_^2^: 0.094**
	B_ASSQ_: 0.33 (η_p_^2^: 0.016; p: 0.18)	B_ASSQ_: 1.8*10^−3^ (η_p_^2^: 0.019; p: 0.14)	B_ASSQ_: −7.5*10^−4^ (η_p_^2^: 0.073; p: 0.004)**
	B_SRS_: −0.2 (η_p_^2^: 0.031; p: 0.062)	B_SRS_: −8.4*10^−4^ (η_p_^2^: 0.023; p: 0.11)	B_SRS_: −5.7*10^−6^ (η_p_^2^: 2.5*10^−4^; p: 0.96)
	B_Age_: 0.11 (η_p_^2^: 6.1*10^−3^; p: 0.41)	B_Age_: 4.7*10^−4^ (η_p_^2^: 4.7*10^−3^; p: 0.47)	B_Age_: −1.5*10^−4^ (η_p_^2^: 0.012; p: 0.25)
	B_viq_: 0.075 (η_p_^2^: 3.2*10^−3^; p: 0.59)	B_viq_: 5.6*10^−4^ (η_p_^2^: 5.8*10^−3^; p: 0.42)	B_viq_: −5.6*10^−4^ (η_p_^2^: 0.12; p: 1.7*10^−4^)***
	B_Gender_: 0.94 (η_p_^2^: 1.4*10^−3^; p: 0.78)	B_Gender_: 3.3*10^−3^ (η_p_^2^: 3.6*10^−4^; p: 0.84)	B_Gender_: 8.7*10^−4^ (η_p_^2^: 5.7*10^−4^; p: 0.8)
Right parahippocampal gyrus, posterior part	**F: 0.19**	**F: 0.56**	**F: 12.29**
	**p: 0.66**	**p: 0.46**	**p: 0.00066** *** †
	**η_p_^2^: 0.002**	**η_p_^2^: 0.005**	**η_p_^2^: 0.101**
	B_ASSQ_: 0.11 (η_p_^2^: 1.3*10^−3^; p: 0.69)	B_ASSQ_: 9.8*10^−4^ (η_p_^2^: 2.8*10^−3^; p: 0.58)	B_ASSQ_: −1.2*10^−3^ (η_p_^2^: 0.088; p: 0.001)**
	B_SRS_: −4.3*10^−4^ (η_p_^2^: ∼0; p: 0.99)	B_SRS_: 1.8*10^−4^ (η_p_^2^: 5.1*10^−4^; p: 0.81)	B_SRS_: 7.1*10^−5^ (η_p_^2^: 1.7*10^−3^; p: 0.66)
	B_Age_: 0.14 (η_p_^2^: 7.8*10^−3^; p: 0.35)	B_Age_: 2.8*10^−4^ (η_p_^2^: 8.2*10^−4^; p: 0.76)	B_Age_: −1.2*10^−4^ (η_p_^2^: 0.003; p: 0.54)
	B_viq_: 0.025 (η_p_^2^: 2.2*10^−4^; p: 0.87)	B_viq_: 7.3*10^−4^ (η_p_^2^: 4.7*10^−3^; p: 0.47)	B_viq_: −3.6*10^−4^ (η_p_^2^: 0.025; p: 0.098)
	B_Gender_: 6.2 (η_p_^2^: 0.022; p: 0.11)	B_Gender_: 0.048 (η_p_^2^: 0.035; p: 0.046)*	B_Gender_: 0.0047 (η_p_^2^: 0.007; p: 0.37)
Left temporal fusiform cortex, posterior part	**F: 0.01**	**F: 0.11**	**F: 11.28**
	**p: 0.93**	**p: 0.74**	**p: 0.0011** ** †
	**η_p_^2^: 6.9** * **10^−5^**	**η_p_^2^: 9.7** * **10^−4^**	**η_p_^2^: 0.093**
	B_ASSQ_: −0.012 (η_p_^2^: 3.1*10^−4^; p: 0.95)	B_ASSQ_: 3.1*10^−4^ (η_p_^2^: 1.1*10^−3^; p: 0.73)	B_ASSQ_: −7.4*10^−4^ (η_p_^2^: 0.073; p: 0.004)**
	B_SRS_: −3.6*10^−3^ (η_p_^2^: 1.7*10^−5^; p: 0.97)	B_SRS_: −4.8*10^−5^ (η_p_^2^: 1.4*10^−4^; p: 0.9)	B_SRS_: −1.8*10^−6^ (η_p_^2^: 3.1*10^−6^; p: 0.99)
	B_Age_: 0.11 (η_p_^2^: 0.01; p: 0.29)	B_Age_: 4.3*10^−4^ (η_p_^2^: 7.6*10^−3^; p: 0.36)	B_Age_: −1.1*10^−4^ (η_p_^2^: 0.006; p: 0.41)
	B_viq_: −0.094 (η_p_^2^: 6.4*10^−3^; p: 0.39)	B_viq_: −2.3*10^−4^ (η_p_^2^: 2*10^−3^; p: 0.64)	B_viq_: −4.3*10^−4^ (η_p_^2^: 0.076; p: 0.003)**
	B_Gender_: −8.2*10^−3^ (η_p_^2^: ∼0; p: 0.99)	B_Gender_: 1.7*10^−4^ (η_p_^2^: 2*10^−7^; p: 0.99)	B_Gender_: 1.3*10^−3^ (η_p_^2^: 1.2*10^−3^; p: 0.71)
Right temporal fusiform cortex, posterior part	**F: 0.1**	**F: 0.32**	**F: 13.46**
	**p: 0.75**	**p: 0.57**	**p: 0.00038** *** †
	**η_p_^2^: 9.1** * **10^−4^**	**η_p_^2^: 0.003**	**η_p_^2^: 0.109**
	B_ASSQ_: 0.14 (η_p_^2^: 3.3*10^−3^; p: 0.54)	B_ASSQ_: 8.9*10^−4^ (η_p_^2^: 5.6*10^−3^; p: 0.43)	B_ASSQ_: −8.7*10^−4^ (η_p_^2^: 0.085; p: 0.002)**
	B_SRS_: −0.076 (η_p_^2^: 5.3*10^−3^; p: 0.45)	B_SRS_: −3.4*10^−4^ (η_p_^2^: 4.5*10^−3^; p: 0.48)	B_SRS_: −6.8*10^−6^ (η_p_^2^: 3.1*10^−5^; p: 0.95)
	B_Age_: 0.043 (η_p_^2^: 1.1*10^−3^; p: 0.73)	B_Age_: 1.8*10^−4^ (η_p_^2^: 8.8*10^−4^; p: 0.76)	B_Age_: 5.9*10^−5^ (η_p_^2^: 1.6*10^−3^; p: 0.67)
	B_viq_: −0.13 (η_p_^2^: 8.3*10^−3^; p: 0.34)	B_viq_: −4.6*10^−4^ (η_p_^2^: 4.7*10^−3^; p: 0.47)	B_viq_: −4.9*10^−4^ (η_p_^2^: 0.083; p: 0.002)**
	B_Gender_: 4.2 (η_p_^2^: 0.016; p: 0.18)	B_Gender_: 0.016 (η_p_^2^: 0.01; p: 0.29)	B_Gender_: 4*10^−4^ (η_p_^2^: 1.1*10^−4^; p: 0.91)

We demonstrate the joint effects of Social Responsiveness Scale (Total Score) and Autism Spectrum Screening Questionnaire, controlling for age and gender and WASI Verbal IQ ( = viq).

* : 0.01≤p<0.05.

** : 0.001≤p<0.01.

*** : p<0.001.

† : Significant interaction after false discovery rate correction with the Benjamini-Hochberg procedure.

**Figure 7:**
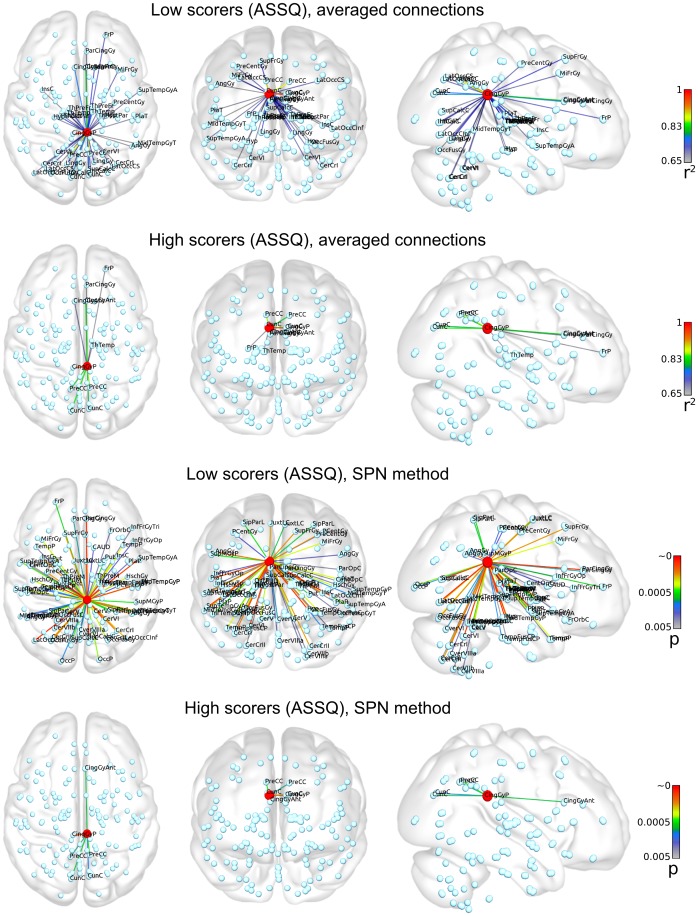
Functional network of the right posterior cingulate cortex, calculated for sub-populations with the lowest and highest ASSQ scores. Graph edges were depicted based on the strongest functional connectivity (threshold criteria for averaged networks: wavelet correlation coefficient >0.634; 95^th^ percentile strongest connection in high scorers; threshold criterion for the statistical parametric network (SPN) method: p<0.005). The nomenclature for brain region abbreviations is given in Table S1. Raw connectivity data are provided in Table 5. First row: averaged functional network (first degree connections) in subjects with the lowest ASSQ score (n = 52; 5th percentile, cut-off threshold: 2). Second row: lowest ASSQ scorer group, SPN based determination of connectivity strengths. Third row: averaged functional network in subjects with the highest ASSQ score (n = 7; 95th percentile, cut-off threshold: 24). Fourth row: high ASSQ scorer group, connections are visualized using SPN analysis.

**Figure 8:**
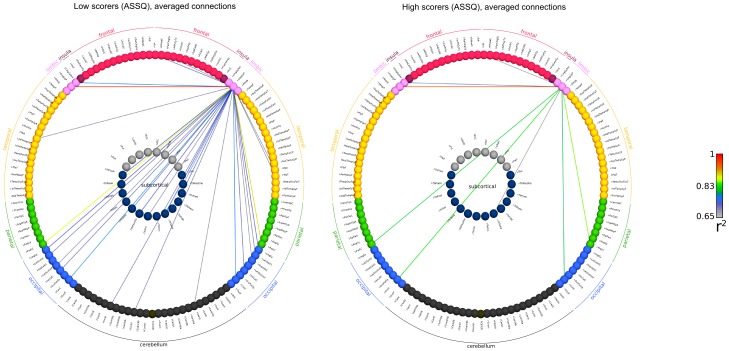
Demonstrating the functional connections of the right posterior cingulate cortex with a circular connectivity profile. Connectivity data were calculated for sub-populations with the lowest and highest ASSQ scores. Key for the abbreviations is given in Table S1.

**Figure 9:**
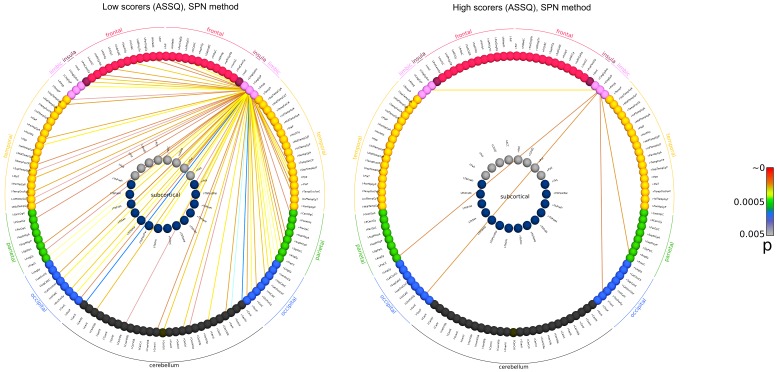
Circular representation of the functional connections of the right posterior cingulate cortex, SPN method. Connectivity data were calculated for sub-populations with the lowest and highest ASSQ scores. Key for the abbreviations is given in Table S1.

An important part of the analysis is to interpret graph theoretical results in a broader context: to relate regional impairments to the brain's overall functional network organization. To portray the sub-network to which the posterior cingulate gyrus belongs, we provide quantitative values for the strengths of first degree connections in this area in Table 5. In subjects with high ASSQ scores, the right posterior cingulate is most strongly connected to its contralateral equivalent and to anterior cingulate gyri in both hemispheres. Further strong connections were found with the precuneus and cuneus cortex, to the temporal connectivity domains of the thalamus and to the right frontal pole. A notable difference is seen in the network structure in low and high scorers of the ASSQ test (the later marking greater traits of autism). For illustrating these networks, we used the correlation coefficient threshold of 0.634 (95^th^ percentile strongest connection), this selected the 9 regions that were most strongly interconnected to the right posterior cingulate gyrus. The same threshold in low scorers revealed 34 connected regions. As the network density is defined by the *wiring cost* [69], correlation coefficients may not be suitable for inter-subject comparisons – we propose to use the statistical parametric network approach. This would help tackle this problem in a way that graph edges (connections) significantly deviating from the group mean can be pinpointed. Using a correlation threshold of p = 0.005 during SPN analysis, a similar difference was noted between high and low ASSQ scorer groups. Using the SPN approach, 6 regions were connected to the right posterior cingulate in high ASSQ scorers, while the same cut-off threshold depicted 75 connections in low ASSQ scorers. This approach reveals an overall reduction of first degree connections of the right posterior cingulate, more specifically, reduced connections to the frontal lobe, superior temporal regions and the occipital lobe. We illustrate the network structure of the right posterior cingulate gyrus in Figure 7, Figure 8 and Figure 9.

**Table 5:** Connections of the right posterior cingulate gyrus in subjects with low and high degree of autistic traits.

Group	Connected brain regions and averaged functional connectivity values (correlation coefficient)
Subjects with the highest ASSQ Total score (n = 52, cut-off value = 2)	**l-CingGyP** (0.91), **r-PreCC** (0.82), **l-CunC** (0.79), **r-CingGyAnt** (0.79), **l-PreCC** (0.78), **r-CunC** (0.76), **l-CingGyAnt** (0.65), **r-ThTemp** (0.63), **r-FrP** (0.63)
Subjects with the lowest ASSQ Total score (n = 7, cut-off value = 24)	**l-CingGyP** (0.94), **r-PreCC** (0.85), **l-PreCC** (0.83), **r-CingGyAnt** (0.78), **l-CunC** (0.75), **l-CingGyAnt** (0.74), **r-CunC** (0.74), **r-ThPreFr** (0.72), **r-ThTemp** (0.72), **l-ThPreFr** (0.71), **l-LingGy** (0.7), **r-SupFrGy** (0.69), **l-ThPostPar** (0.69), **r-LingGy** (0.69), **r-FrP** (0.68), **l-IntCalC** (0.68), **l-ThTemp** (0.67), **r-IntCalC** (0.67), **l-LatOccCS** (0.66), **r-PreCentGy** (0.66), **r-MiFrGy** (0.66), **l-CerVI** (0.66), **l-CerCrI** (0.66), **r-LatOccCS** (0.66), **l-ThOcc** (0.65), **l-LatOccCInf** (0.65), **l-Hyp** (0.65), **r-ParCingGy** (0.65), **r-ThPostPar** (0.64), **r-SupTempGyA** (0.64), **r-CerVI** (0.64), **r-SupCalcC** (0.64), **r-Hyp** (0.64), **r-PlaT** (0.69)

Connection strengths are given as the wavelet correlation coefficients of the BOLD time curves. Cut-off threshold for lowest and highest ASSQ scorers were the 5^th^ and 95^th^ percentile values. Regions were listed in descending order of the connection strengths. Key for the abbreviations is given in Table S1.

## Discussion

### Global functional network correlates of autistic traits

Network-based approaches are valuable for autism research: they allow interpreting brain connectivity information as complex interactions between remote neuronal groups and therefore provide feasible means to study the effects of autism globally. In the graph theoretical model each network node is interconnected with a large number of brain regions, such connections are weighted by the degree of temporal coherence of low-frequency BOLD signals. Hence if a pathological process selectively damages functionally associated groups of neurons, the largest effect will presumably be observed in the region or subgraph having the highest density of connections to the affected areas (i.e. connector hubs). Similarly, generalized impairments in connections by pathologic processes can change the nature of information transfer globally, leaving their mark on graph theoretical descriptors such as global efficiency or degree [81]. In our study the association of regional network efficiency was only significant in the right posterior cingulate gyrus after adjusting for the false discovery rate. In contrast to this observation, reduced average local efficiency (E_l_) in the entire network (averaged over 149 regions) was weakly associated with the autistic trait (η_p_^2^ = 0.047; F = 5.99; p = 0.016). This result can putatively be ascribed to the summation of impairments in distributed regions that are not significant after the relatively conservative FDR adjustment (Table S2). Recently, Dennis and colleagues provided whole-brain measures for the association of structural network structure and the presence of rs2710102 CNTNAP2 autism risk gene [82]. A significant reduction of global efficiency, small-worldness and elevated characteristic path length was discovered in carriers of the gene. Using EEG data, Barttfeld et al. showed that the functional network of subjects with autism spectrum disorder have less clustering coefficient and increased characteristic path length than controls [83].

### Regional functional network correlates of autistic traits

According to our functional network analysis, the posterior cingulate gyrus was affected in both hemispheres while we revealed significantly decreased network degree and efficiency for the right posterior cingulate cortex. The cingulate region was already proved to play a role in the decreased inhibitory control in high-functioning autists [84]. Additional support comes from a study describing neuronal migrational disorder in the cingulate cortex [85], although the case report by Korkmaz and co-authors does not imply that such a migration deficit could represent common pathogenic pathway for autism spectrum disorders. A histological analysis of autistic brains by Oblak and colleagues showed that the PCC had altered cytoarchitecture with irregularly distributed neurons, poorly demarcated layers IV and V, and increased presence of white matter neurons [86]. The possible role of the posterior cingulate in the manifestation of adverse socio-emotional behaviors was postulated: reduced GABA(A) receptors were found in this region and also in the fusiform gyrus [87]. By explicating the neural correlates of moral reasoning in ASD, Schneider et al. revealed decreased activation in the amygdala while an increase was seen in the cingulate gyri [88]. When we controlled the extended functional network of the posterior cingulate gyrus, first degree connections were found with the cuneus and precuneus cortex, dorsolateral prefrontal cortex (DLPFC) and superior temporal areas (Table 5, Figure 7). This pattern coincides with the common descriptions of the task negative network ( = TNN) [89]. The TNN is known to activate during performance of emotional, social and introspective tasks, such as the theory of mind [90], social perception, emotional processing, viewing familiar faces [91] and its impairments have been frequently linked with the autism spectrum disorder. Even though there is a considerable uncertainty about the function of the posterior cingulate cortex, functional neuroimaging studies postulate it as a cortical hub and assume a role in regulating the balance between internally and externally directed cognition [92]. Intrinsically defined maps of the TNN were found to be altered in autists [93]. Kennedy and co-authors showed that the posterior cingulate cortex and precuneus are reproducibly included in TNN in autistic subjects while other components are diminished, although this effect was only significant for the medial prefrontal cortex and the left angular gyrus. The lowered temporal synchrony of such interconnected areas of the TNN, including the posterior cingulate, can result in reduced network degree or nodal efficiency which is coherent with our findings. The limbic system including the cingulate gyrus is closely tied to emotion and social behaviors and it is an almost trivial statement that the disrupted limbic circuitry could provoke some of the behavioral deficits seen in autists [94].

A review by Courchesne et al. postulates that frontal lobe abnormality is to be held responsible for the social, emotional and cognitive impairments in autism [29]. This is supported by observations of early localized enlargement of the frontal lobe in young children [95] and impaired frontal activation in tasks evaluating selective attention [96] or language skills [97]. In contrast to the commonly described frontal abnormality linked with the autistic trait, we were unable to show such a pattern. Interestingly, the temporal connectivity domain of the thalamus (as defined by the Oxford Thalamus connectivity atlas) was found to be among the first degree connections of the right posterior cingulate gyrus and hence its influence on the network efficiency can be assumed. The thalamus plays a role in cognitive processes by relaying cortico-striato-thalamo-cortical loops: three of such circuits originate from the prefrontal (dorsolateral prefrontal and medial orbitofrontal cortex) and limbic (anterior cingulate cortex) cortical areas and pass through the mediodorsal thalamic nucleus [98]. This model was further stratified by in vivo neuroimaging studies mapping such neuronal pathways [99] and revealing correlations between the thalamic representation of such circuits and executive functions of human subjects [100]. In the present study we have included the thalamus and various other subcortical territories in the construction of the functional network. Although we did not reveal localized disruption in network topology of the thalamus, it occurs that the autism spectrum disorder as a distributed disease could affect the thalamus [101], [102] and the basal ganglia as well [103].

People with autism spectrum disorder exhibit heterogeneous social-communication characteristics, a phenotype that presumably extends into the neurotypical population [104]. So far, we reported our findings in comparison to the studies using clinical (low or high functioning autistic) populations. A different design of investigations was necessary to find evidence about such autistic traits in normally developed subjects. In a study by Hagen et al. structural and functional impairment of superior temporal sulcus (pSTS) was implicated to correlate with self-reported traits autism spectrum characteristics [105]. Similarly to our study, Di Martino et al. described the neural correlates of the autistic traits in neurotypical populations and revealed reduced cingulo-insular functional connectivity in resting state functional imaging data [37]. Significant negative relationship of this specific connection was found with the SRS score which is partially coherent with our findings, however, currently we do not report the effects on edges ( = individual connections) but provide descriptors of network topology.

### White matter impairments linked with autistic traits

Measuring functional network topology and regional diffusion anisotropy in the same autistic subject group can provide complementary information on a more pervasive structural deficit that is characterized by the disruption of white matter integrity, altered connections or conversely, increased anisotropy could indirectly indicate “more ordered” axons compared to the control group [106]. In both hemispheric temporal fusiform gyri, we have revealed diffusion anisotropy deficits. A mixed pattern of elevated and reduced FA values was reported by Cheng and colleagues [19]. Although individuals affected by autistic spectrum disorders do not usually show severe behavioral consequences of prosopagnosia, abnormal response to face stimuli was reported [107], [108], reduced functional connectivity from the fusiform face area (FFA) and its associated networks was shown [109], but conversely, the role of FFA impairment in autism was doubted by others [110]. Moreover, neuropathological evaluations discovered microscopic structural deficits in this territory, smaller neuronal volumes and densities were found [111]. Besides the diffusion abnormalities of the fusiform gyrus, we showed the bilateral association of the FA values of the parahippocampal gyrus and the self-reported autistic traits. Among distributed pattern of cortical regions, the cortical thickness of the left parahippocampal gyrus was found to be a predictor of autism spectrum disorders [112]. In contrast to this observation, Ke and colleagues linked the reduction of the right parahippocampal gray matter volume with HFA [113]. The significance of the posterior cingulate cortex in autism spectrum disorders was also noted by Uddin et al. who used multivariate pattern analysis to find the most discriminative features between normal subjects and ASD. The largest discriminative power was achieved by using gray matter thickness measurements in the posterior cingulate cortex, the parahippocampal gyri and the anterior temporal lobe [114].

### Limitations and future directions

A major limitation in our study protocol is that neither the ASSQ nor the SRS provides explicit diagnostic data on the autism spectrum disorder including HFA. Furthermore, the diagnostic border between Asperger's syndrome and HFA is not well-defined; it is acknowledged that the key to the diagnosis is the presence of early language developmental impairment. We did not have such historic information about the adult subjects involved. In conclusion, our results can only be interpreted as the effects of the self-reported autistic trait on the functional brain connectome and diffusion anisotropy patterns, observed in normally functioning adults. We raised the specificity of our study by employing a conservative FDR adjustment method, although many of our measurements are highly dependent from each other; for instance, hemispheric equivalent regions are highly synchronized (r>0.9). Therefore the decision threshold in terms of accepted p-values could be higher and impairments in a larger set of regions would be linked to the autistic traits. Furthermore, by building and analyzing larger and clinically controlled datasets such as the ABIDE ( = Autism Brain Imaging Data Exchange), it will become possible to explicate the neural correlates of this puzzling mental condition while more plausible biomarkers could be developed for the diagnostics and prognostics of ASD.

### Conclusions

Recent large-scale neuroimaging data-sharing initiatives such as the INDI provided valuable normative functional imaging resources for discovery research. Such complex datasets allow the observation of the relationship between brain structure and dimensional psychological scales in neurotypicals. One such challenging task is exploring traits with relatively low prevalence like autism. We conclude that high functioning autistic adults carry peculiar brain abnormalities that are linked with regionally disrupted functional networks and altered regional diffusion anisotropy of the brain, especially in the limbic cortex while reduced diffusion anisotropy is present in temporal fusiform and parahippocampal gyri. A general tendency was observed towards lower averaged local efficiency in subjects with higher scores of self-reported ASSQ and SRS. In contrast to studies revealing frontal and temporal abnormalities in autism, we did not reveal such effects in neurotypical adults with different degrees of autistic traits.

## Supporting Information

Table S1 **Description of the custom brain region system used for creating network nodes and regions of the diffusion anisotropy analysis.**
(DOCX)

Table S2 **Regional neural correlates of self-reported autistic traits, unadjusted F and p values for all brain regions.**
(XLS)

Document S1 **Pseudo code of cost integration method used for calculating the global and regional graph theoretical network descriptors.**
(PDF)

Document S2 **Scripts used in SPSS to perform GLM analysis and false discovery rate adjustment.**
(DOCX)

## References

[1] APA (2007) Diagnostic and Statistical Manual of Mental Disorders, Fourth Edition, Text Revision. Washington DC: American Psychiatric Association.

[2] Baron-Cohen S, Jolliffe T, Mortimore C, Robertson M (1997) Another advanced test of theory of mind: evidence from very high functioning adults with autism or Asperger Syndrome. J Child Psychol Psychiatry 38: 813–822. 9363580 10.1111/j.1469-7610.1997.tb01599.x

[3] Bauman ML, Kemper TL (2005) Neuroanatomic observations of the brain in autism: a review and future directions. Int J Dev Neurosci 23: 183–187. 15749244 10.1016/j.ijdevneu.2004.09.006

[4] Aylward EH, Minshew NJ, Field K, Sparks BF, Singh N (2002) Effects of age on brain volume and head circumference in autism. Neurology 59: 175–183. 12136053 10.1212/wnl.59.2.175

[5] Courchesne E, Pierce K, Schumann CM, Redcay E, Buckwalter JA, et al (2007) Mapping early brain development in autism. Neuron 56: 399–413. 17964254 10.1016/j.neuron.2007.10.016

[6] Riva D, Bulgheroni S, Aquino D, Di Salle F, Savoiardo M, et al (2011) Basal forebrain involvement in low-functioning autistic children: a voxel-based morphometry study. Am J Neuroradiol 32: 1430–1435. 21700792 10.3174/ajnr.A2527 PMC7964346

[7] Ecker C, Suckling J, Deoni SC, Lombardo MV, Bullmore ET, et al (2012) Brain anatomy and its relationship to behavior in adults with autism spectrum disorder: a multicenter magnetic resonance imaging study. Arch Gen Psychiatry 69: 195–209. 22310506 10.1001/archgenpsychiatry.2011.1251

[8] Greimel E, Nehrkorn B, Schulte-Rüther M, Fink GR, Nickl-Jockschat T, et al (2012) Changes in grey matter development in autism spectrum disorder. Brain Struct Funct [Epub ahead of print] DOI:10.1007/s00429-012-0439-9. 10.1007/s00429-012-0439-9 PMC3695319 22777602

[9] Scheel C, Rotarska-Jagiela A, Schilbach L, Lehnhardt FG, Krug B, et al (2011) Imaging derived cortical thickness reduction in high-functioning autism: key regions and temporal slope. Neuroimage 58: 391–400. 21749926 10.1016/j.neuroimage.2011.06.040

[10] Hyde KL, Samson F, Evans AC, Mottron L (2010) Neuroanatomical differences in brain areas implicated in perceptual and other core features of autism revealed by cortical thickness analysis and voxel-based morphometry. Hum Brain Mapp 31: 556–566. 19790171 10.1002/hbm.20887 PMC6870833

[11] Yu KK, Cheung C, Chua SE, McAlonan GM (2011) Can Asperger syndrome be distinguished from autism? An anatomic likelihood meta-analysis of MRI studies. J Psychiatry Neurosci 36: 412–421. 21406158 10.1503/jpn.100138 PMC3201995

[12] Kwon H, Ow AW, Pedatella KE, Lotspeich LJ, Reiss AL (2004) Voxel-based morphometry elucidates structural neuroanatomy of high-functioning autism and Asperger syndrome. Dev Med Child Neurol 46: 760–764. 15540637 10.1017/s0012162204001306

[13] Le Bihan D, Johansen-Berg H (2011) Diffusion MRI at 25: Exploring brain tissue structure and function. Neuroimage 61: 324–341. 22120012 10.1016/j.neuroimage.2011.11.006 PMC3683822

[14] Kubicki M, Westin CF, Maier SE, Mamata H, Frumin M, et al (2002) Diffusion tensor imaging and its application to neuropsychiatric disorders. Harv Rev Psychiatry 10: 324–336. 12485979 10.1080/10673220216231 PMC2853779

[15] Moseley M, Bammer R, Illes J (2002) Diffusion-tensor imaging of cognitive performance. Brain Cogn 50: 396–413. 12480486 10.1016/s0278-2626(02)00524-9

[16] Barnea-Goraly N, Kwon H, Menon V, Eliez S, Lotspeich L, et al (2004) White matter structure in autism: preliminary evidence from diffusion tensor imaging. Biol Psychiatry 55: 323–326. 14744477 10.1016/j.biopsych.2003.10.022

[17] Sundaram SK, Kumar A, Makki MI, Behen ME, Chugani HT, et al (2008) Diffusion tensor imaging of frontal lobe in autism spectrum disorder. Cereb Cortex 18: 2659–2665. 18359780 10.1093/cercor/bhn031 PMC2567426

[18] Bloemen OJ, Deeley Q, Sundram F, Daly EM, Barker GJ, et al (2010) White matter integrity in Asperger syndrome: a preliminary diffusion tensor magnetic resonance imaging study in adults. Autism Res 3: 203–213. 20625995 10.1002/aur.146

[19] Cheng Y, Chou KH, Chen IY, Fan YT, Decety J, et al (2010) Atypical development of white matter microstructure in adolescents with autism spectrum disorders. Neuroimage 50: 873–882. 20074650 10.1016/j.neuroimage.2010.01.011

[20] Sahyoun CP, Belliveau JW, Mody M (2010) White matter integrity and pictorial reasoning in high-functioning children with autism. Brain Cogn 73: 180–818. 20542370 10.1016/j.bandc.2010.05.002 PMC2905578

[21] Just MA, Cherkassky VL, Keller TA, Kana RK, Minshew NJ (2007) Functional and anatomical cortical underconnectivity in autism: evidence from an fMRI study of an executive function task and corpus callosummorphometry. Cerebral Cortex 17: 951–961. 16772313 10.1093/cercor/bhl006 PMC4500121

[22] Solomon M, Ozonoff SJ, Ursu S, Ravizza S, Cummings N, et al (2009) The neural substrates of cognitive control deficits in autism spectrum disorders. Neuropsychologia 47: 2515–2526. 19410583 10.1016/j.neuropsychologia.2009.04.019 PMC2766616

[23] Jones TB, Bandettini PA, Kenworthy L, Case LK, Milleville SC, et al (2010) Sources of group differences in functional connectivity: an investigation applied to autism spectrum disorder. Neuroimage 49: 401–414. 19646533 10.1016/j.neuroimage.2009.07.051 PMC2832835

[24] Damoiseaux JS, Rombouts SA, Barkhof F, Scheltens P, Stam CJ, et al (2006) Consistent resting-state networks across healthy subjects. Proc Natl Acad Sci U S A 103: 13848–13853. 16945915 10.1073/pnas.0601417103 PMC1564249

[25] Greicius MD, Krasnow B, Reiss AL, Menon V (2003) Functional connectivity in the resting brain: a network analysis of the default mode hypothesis. Proc Natl Acad Sci U S A 100: 253–258. 12506194 10.1073/pnas.0135058100 PMC140943

[26] Cole DM, Smith SM, Beckmann CF (2010) Advances and pitfalls in the analysis and interpretation of resting-state FMRI data. Front Syst Neurosci 4: 8. 20407579 10.3389/fnsys.2010.00008 PMC2854531

[27] Guye M, Bettus G, Bartolomei F, Cozzone PJ (2010) Graph theoretical analysis of structural and functional connectivity MRI in normal and pathological brain networks. Magn Reson Mater Phy 23: 409–421. 10.1007/s10334-010-0205-z 20349109

[28] Bullmore E, Sporns O (2009) Complex brain networks: graph theoretical analysis of structural and functional systems. Nat Rev Neurosci 10: 186–198. 19190637 10.1038/nrn2575

[29] Courchesne E, Pierce K (2005) Why the frontal cortex in autism might be talking only to itself: local over-connectivity but long-distance disconnection. Curr Opin Neurobiol 15: 225–230. 15831407 10.1016/j.conb.2005.03.001

[30] Noonan SK, Haist F, Müller RA (2009) Aberrant functional connectivity in autism: evidence from low-frequency BOLD signal fluctuations. Brain Res 1262: 48–63. 19401185 10.1016/j.brainres.2008.12.076 PMC2766184

[31] Shih P, Shen M, Ottl B, Keehn B, Gaffrey MS, et al (2010) Atypical network connectivity for imitation in autism spectrum disorder. Neuropsychologia 48: 2931–2939. 20558187 10.1016/j.neuropsychologia.2010.05.035 PMC3315839

[32] Ke X, Tang T, Hong S, Hang Y, Zou B, et al (2009) White matter impairments in autism, evidence from voxel-based morphometry and diffusion tensor imaging. Brain Res 1265: 171–177. 19233148 10.1016/j.brainres.2009.02.013

[33] Wing L (1988) The continuum of autistic characteristics. In: Schopler E, Mesibov GB, editors. Diagnosis and assessment in autism.Current issues in autism. New York, NY, US: Plenum Press. 91–110 pp.

[34] Nooner KB, Colcombe SJ, Tobe RH, Mennes M, Benedict MM, et al (2012) The NKI-Rockland Sample: A Model for Accelerating the Pace of Discovery Science in Psychiatry. Front Neurosci 6: 152 doi:10.3389/fnins.2012.00152. 23087608 10.3389/fnins.2012.00152 PMC3472598

[35] Ehlers S, Gillberg C, Wing L (1999) A Screening Questionnaire for Asperger Syndrome and Other High Functioning Autism Spectrum Disorders in School Age Children. J Autism Dev Disord 29: 129–141. 10382133 10.1023/a:1023040610384

[36] Constantino JN, Davis SA, Todd RD, Schindler MK, Gross MM, et al (2003) Validation of a brief quantitative measure of autistic traits: comparison of the social responsiveness scale with the autism diagnostic interview revised. J Autism Dev Disord 33: 427–433. 12959421 10.1023/a:1025014929212

[37] Di Martino A, Shehzad Z, Kelly C, Roy AK, et al (2009) Relationship between cingulo-insular functional connectivity and autistic traits in neurotypical adults. Am J Psychiatry 166: 891–899. 19605539 10.1176/appi.ajp.2009.08121894 PMC3075727

[38] Siegel DJ, Minshew NJ, Goldstein G (1996) Wechsler IQ profiles in diagnosis in high-functioning autism. J Autism Dev Disord 26: 389–406. 8863091 10.1007/BF02172825

[39] Williams DL, Goldstein G, Kojkowski N, Minshew NJ (2008) Do individuals with high functioning autism have the IQ profile associated with nonverbal learning disability? Res Autism Spectr Disord 2: 353–361. 18516234 10.1016/j.rasd.2007.08.005 PMC2394183

[40] Wechsler D (1999) Wechsler Abbreviated Scale of Intelligence. San Antonio, TX: The Psychological Corporation.

[41] Jenkinson M, Beckmann CF, Behrens TE, Woolrich MW, Smith SM (2012) Fsl. Neuroimage 62: 782–790. 21979382 10.1016/j.neuroimage.2011.09.015

[42] Smith SM, Jenkinson M, Woolrich MW, Beckmann CF, Behrens TEJ, et al (2004) Advances in functional and structural MR image analysis and implementation as FSL. NeuroImage 23: 208–219. 10.1016/j.neuroimage.2004.07.051 15501092

[43] Woolrich MW, Jbabdi S, Patenaude B, Chappell M, Makni S, et al (2009) Bayesian analysis of neuroimaging data in FSL. NeuroImage 45: S173–186. 19059349 10.1016/j.neuroimage.2008.10.055

[44] Jenkinson M, Beckmann CF, Behrens TEJ, Woolrich MW, Smith SM (2012) FSL. NeuroImage 62: 782–790. 21979382 10.1016/j.neuroimage.2011.09.015

[45] Greve DN, Fischl B (2009) Accurate and robust brain image alignment using boundary-based registration. NeuroImage 48: 63–72. 19573611 10.1016/j.neuroimage.2009.06.060 PMC2733527

[46] Jenkinson M, Smith SM (2001) A Global Optimisation Method for Robust Affine Registration of Brain Images. Medical Image Analysis 5: 143–156. 11516708 10.1016/s1361-8415(01)00036-6

[47] Jenkinson M, Bannister P, Brady JM, Smith SM (2002) Improved Optimisation for the Robust and Accurate Linear Registration and Motion Correction of Brain Images. NeuroImage 17: 825–841. 12377157 10.1016/s1053-8119(02)91132-8

[48] Smith SM (2002) Fast robust automated brain extraction. Hum Brain Mapp 17: 143–155. 12391568 10.1002/hbm.10062 PMC6871816

[49] Fonov VS, Evans AC, McKinstry RC, Almli CR, Collins DL (2009) Unbiased nonlinear average age-appropriate brain templates from birth to adulthood. NeuroImage 47 S1: 102 doi:10.1016/S1053-8119%2809%2970884-5.

[50] Fonov VS, Evans AC, Botteron K, Almli CR, McKinstry RC, et al (2011) Unbiased average age-appropriate atlases for pediatric studies. NeuroImage 54: 313–327. 20656036 10.1016/j.neuroimage.2010.07.033 PMC2962759

[51] Smith SM, Miller KL, Salimi-Khorshidi G, Webster M, Beckmann CF, et al (2011) Network modelling methods for FMRI. Neuroimage 54: 875–891. 20817103 10.1016/j.neuroimage.2010.08.063

[52] Frazier JA, Chiu S, Breeze JL, Makris N, Lange N, et al (2005) Structural brain magnetic resonance imaging of limbic and thalamic volumes in pediatric bipolar disorder. Am J Psychiatry 162: 1256–1265. 15994707 10.1176/appi.ajp.162.7.1256

[53] Desikan RS, Ségonne F, Fischl B, Quinn BT, Dickerson BC, et al (2006) An automated labeling system for subdividing the human cerebral cortex on MRI scans into gyral based regions of interest. Neuroimage 31: 968–980. 16530430 10.1016/j.neuroimage.2006.01.021

[54] Makris N, Goldstein JM, Kennedy D, Hodge SM, Caviness VS, et al (2006) Decreased volume of left and total anterior insular lobule in schizophrenia. Schizophr Res 83: 155–171. 16448806 10.1016/j.schres.2005.11.020

[55] Goldstein JM, Seidman LJ, Makris N, Ahern T, O'Brien LM, et al (2007) Hypothalamic abnormalities in schizophrenia: sex effects and genetic vulnerability. Biol Psychiatry 61: 935–945. 17046727 10.1016/j.biopsych.2006.06.027

[56] Diedrichsen J, Balsters JH, Flavell J, Cussans E, Ramnani N (2009) A probabilistic MR atlas of the human cerebellum. Neuroimage 46: 39–46. 19457380 10.1016/j.neuroimage.2009.01.045

[57] Behrens TE, Johansen-Berg H, Woolrich MW, Smith SM, Wheeler-Kingshott CA, et al (2003) Non-invasive mapping of connections between human thalamus and cortex using diffusion imaging. Nat Neurosci 6: 750–757. 12808459 10.1038/nn1075

[58] Behrens TEJ, Woolrich MW, Jenkinson M, Johansen-Berg H, Nunes RG (2003) Characterisation and propagation of uncertainty in diffusion weighted MR imaging. Magnetic Resonance in Medicine 50: 1077–1088. 14587019 10.1002/mrm.10609

[59] Basser PJ, Pierpaoli C (1996) Microstructural and physiological features of tissues elucidated by quantitative-diffusion-tensor MRI. J Magn Reson B 111: 209–219. 8661285 10.1006/jmrb.1996.0086

[60] Skidmore F, Korenkevych D, Liu Y, He G, Bullmore E, et al (2011) Connectivity brain networks based on wavelet correlation analysis in Parkinson fMRI data. Neuroscience Letters 499: 47–51. 21624430 10.1016/j.neulet.2011.05.030

[61] Cordes D, Haughton VM, Arfanakis K, Carew JD, Turski PA, et al (2001) Frequencies contributing to functional connectivity in the cerebral cortex in “resting-state” data. AJNR Am J Neuroradiol 22: 1326–1333. 11498421 PMC7975218

[62] Achard S, Bassett DS, Meyer-Lindenberg A, Bullmore ET (2008) Fractal connectivity of long-memory networks. Physical Review E 77: 036104. 10.1103/PhysRevE.77.036104 18517458

[63] Percival DB, Walden AT (2000) Wavelet Methods for Time Series Analysis, Cambridge: Cambridge University Press. 622 p.

[64] Achard S, Salvador R, Whitcher B, Suckling J, Bullmore ET (2006) A resilient, low-frequency, small-world human brain functional network with highly connected association cortical hubs. J Neurosci 26: 63–72. 16399673 10.1523/JNEUROSCI.3874-05.2006 PMC6674299

[65] Barrat A, Barthélemy M, Pastor-Satorras R, Vespignani A (2004) The architecture of complex weighted networks. Proc Natl Acad Sci U S A 101: 3747–52. 15007165 10.1073/pnas.0400087101 PMC374315

[66] Newman MEJ (2003) The structure and function of complex networks. SIAM Review 45: 167–256.

[67] Rubinov M, Sporns O (2010) Complex network measures of brain connectivity: uses and interpretations. Neuroimage 52: 1059–1069. 19819337 10.1016/j.neuroimage.2009.10.003

[68] Horvath S, Dong J (2008) Geometric interpretation of gene coexpression network analysis. PLoS Comput Biol 4: e1000117. 18704157 10.1371/journal.pcbi.1000117 PMC2446438

[69] Ginestet CE, Nichols TE, Bullmore ET, Simmons A (2011) Brain network analysis: separating cost from topology using cost-integration. PLoS One 6: e21570. 21829437 10.1371/journal.pone.0021570 PMC3145634

[70] Latora V, Marchiori M (2001) Efficient Behavior of Small-World Networks,. Phys Rev Lett 87: 198701. 11690461 10.1103/PhysRevLett.87.198701

[71] Basset DS, Bullmore E (2006) Small-world brain networks. Neuroscientist 12: 512–523. 17079517 10.1177/1073858406293182

[72] Crucitti P, Latora V, Marchiori M, Rapisarda A (2003) Efficiency of Scale-Free Networks: Error and Attack Tolerance. Physica A 320: 642 doi:10.1016/S0378-4371(02)01545-5.

[73] Floyd RW (1962) Algorithm 97: Shortest path. Comm ACM 5: 345 DOI:10.1145/367766.368168.

[74] Dijkstra EW (1959) A note on two problems in connexion with graphs. Numerische Mathematik 1: 269–271.

[75] Bullmore E, Sporns O (2012) The economy of brain network organization. Nat Rev Neurosci 13: 336–349. 22498897 10.1038/nrn3214

[76] Schindler KA, Bialonski S, Horstmann M, Elger CE, Lehnertz K (2008) Evolving functional network properties and synchronizability during human epileptic seizures. Chaos 18: 033119 doi:10.1063/1.2966112. 19045457 10.1063/1.2966112

[77] Ginestet CE, Simmons A (2011) Statistical Parametric Network Analysis of Functional Connectivity Dynamics during a Working Memory Task. Neuroimage 55: 688–704. 21095229 10.1016/j.neuroimage.2010.11.030

[78] Cohen J (1988) Statistical power analysis for the behavioral sciences (2nd ed.). Hillsdale: Erlbaum.

[79] Benjamini Y, Hochberg Y (1995) Controlling the false discovery rate: a practical and powerful approach to multiple testing. Journal of the Royal Statistical Society, Series B (Methodological) 57: 289–300.

[80] Achard S, Bullmore E (2007) Efficiency and Cost of Economical Brain Functional Networks. PLoS Comput Biol 3: e17 doi:10.1371/journal.pcbi.0030017. 17274684 10.1371/journal.pcbi.0030017 PMC1794324

[81] Cabral J, Kringelbach ML, Deco G (2012) Functional graph alterations in schizophrenia: a result from a global anatomic decoupling? Pharmacopsychiatry 45 Suppl 1: S57–64. 22565236 10.1055/s-0032-1309001

[82] Dennis EL, Jahanshad N, Rudie JD, Brown JA, Johnson K, et al (2011) Altered structural brain connectivity in healthy carriers of the autism risk gene, CNTNAP2. Brain Connect 1: 447–359. 22500773 10.1089/brain.2011.0064 PMC3420970

[83] Barttfeld P, Wicker B, Cukier S, Navarta S, Lew S, et al (2011) A big-world network in ASD: dynamical connectivity analysis reflects a deficit in long-range connections and an excess of short-range connections. Neuropsychologia 49: 254–263. 21110988 10.1016/j.neuropsychologia.2010.11.024

[84] Kana RK, Keller TA, Minshew NJ, Just MA (2007) Inhibitory control in high-functioning autism: decreased activation and underconnectivity in inhibition networks. Biol Psychiatry 62: 198–206. 17137558 10.1016/j.biopsych.2006.08.004 PMC4492460

[85] Korkmaz B, Benbir G, Demirbilek V (2006) Migration abnormality in the left cingulate gyrus presenting with autistic disorder. J Child Neurol 21: 600–604. 16970852 10.1177/08830738060210070601

[86] Oblak AL, Rosene DL, Kemper TL, Bauman ML, Blatt GJ (2011) Altered posterior cingulate cortical cyctoarchitecture, but normal density of neurons and interneurons in the posterior cingulate cortex and fusiform gyrus in autism. Autism Res 4: 200–211. 21360830 10.1002/aur.188 PMC3110607

[87] Oblak AL, Gibbs TT, Blatt GJ (2011) Reduced GABAA receptors and benzodiazepine binding sites in the posterior cingulate cortex and fusiform gyrus in autism. Brain Res 1380: 218–228. 20858465 10.1016/j.brainres.2010.09.021 PMC3020259

[88] Schneider K, Pauly KD, Gossen A, Mevissen L, Michel TM, et al (2012) Neural correlates of moral reasoning in autism spectrum disorder. Soc Cogn Affect Neurosci DOI:10.1093/scan/nss051. 10.1093/scan/nss051 PMC3739915 22569187

[89] Raichle ME, Snyder AZ (2007) A default mode of brain function: a brief history of an evolving idea. Neuroimage 37: 1083–1090. 17719799 10.1016/j.neuroimage.2007.02.041

[90] Vogeley K, Bussfeld P, Newen A, Herrmann S, Happé F, et al (2001) Mind reading: neural mechanisms of theory of mind and self-perspective. Neuroimage 14: 170–181. 11525326 10.1006/nimg.2001.0789

[91] Pierce K, Haist F, Sedaghat F, Courchesne E (2004) The brain response to personally familiar faces in autism: findings of fusiform activity and beyond. Brain 127: 2703–2716. 15319275 10.1093/brain/awh289

[92] Leech R, Braga R, Sharp DJ (2012) Echoes of the brain within the posterior cingulate cortex. J Neurosci 32: 215–222. 22219283 10.1523/JNEUROSCI.3689-11.2012 PMC6621313

[93] Kennedy DP, Courchesne E (2008) The intrinsic functional organization of the brain is altered in autism. Neuroimage 39: 1877–1885. 18083565 10.1016/j.neuroimage.2007.10.052

[94] Sokolowski K, Corbin JG (2012) Wired for behaviors: from development to function of innate limbic system circuitry. Front Mol Neurosci 5: 55. 22557946 10.3389/fnmol.2012.00055 PMC3337482

[95] Carper R, Courchesne E (2005) Localized enlargement of the frontal lobe in autism. Biol Psychiatry 57: 126–133. 15652870 10.1016/j.biopsych.2004.11.005

[96] Belmonte MK, Yurgelun-Todd DA (2003) Functional anatomy of impaired selective attention and compensatory processing in autism. Brain Res Cogn Brain Res 17: 651–664. 14561452 10.1016/s0926-6410(03)00189-7

[97] Just MA, Cherkassky VL, Keller TA, Minshew NJ (2004) Cortical activation and synchronization during sentence comprehension in high-functioning autism: evidence of underconnectivity. Brain 127: 1811–1121. 15215213 10.1093/brain/awh199

[98] Alexander GE, DeLong MR, Strick PL (1986) Parallel organization of functionally segregated circuits linking basal ganglia and cortex. Annu Rev Neurosci 9: 357–381. 3085570 10.1146/annurev.ne.09.030186.002041

[99] Draganski B, Kherif F, Klöppel S, Cook PA, Alexander DC, et al (2008) Evidence for segregated and integrative connectivity patterns in the human Basal Ganglia. J Neurosci 28: 7143–7152. 18614684 10.1523/JNEUROSCI.1486-08.2008 PMC6670486

[100] Jakab A, Blanc R, Berényi EL (2012) Mapping changes of in vivo connectivity patterns in the human mediodorsal thalamus: correlations with higher cognitive and executive functions. Brain Imaging Behav 6: 472–483. 22584775 10.1007/s11682-012-9172-5

[101] Tsatsanis KD, Rourke BP, Klin A, Volkmar FR, Cicchetti D, et al (2003) Reduced thalamic volume in high-functioning individuals with autism. Biol Psychiatry 53: 121–129. 12547467 10.1016/s0006-3223(02)01530-5

[102] Hardan AY, Minshew NJ, Melhem NM, Srihari S, Jo B, et al (2008) An MRI and proton spectroscopy study of the thalamus in children with autism. Psychiatry Res 163: 97–105. 18508243 10.1016/j.pscychresns.2007.12.002 PMC2467447

[103] Estes A, Shaw DW, Sparks BF, Friedman S, Giedd JN, et al (2011) Basal ganglia morphometry and repetitive behavior in young children with autism spectrum disorder. Autism Res 4: 212–220. 21480545 10.1002/aur.193 PMC3110551

[104] Baron-Cohen S, Wheelwright S, Hill J, Raste Y, Plumb I (2001) The “Reading the Mind in the Eyes” Test revised version: a study with normal adults, and adults with Asperger syndrome or high-functioning autism. J Child Psychol Psychiatry 42: 241–251. 11280420

[105] von dem Hagen EA, Nummenmaa L, Yu R, Engell AD, Ewbank MP, et al (2011) Autism spectrum traits in the typical population predict structure and function in the posterior superior temporal sulcus. Cereb Cortex 21: 493–500. 20439317 10.1093/cercor/bhq062 PMC3041005

[106] Zhang H, Hubbard PL, Parker GJ, Alexander DC (2011) Axon diameter mapping in the presence of orientation dispersion with diffusion MRI. Neuroimage 56: 1301–1315. 21316474 10.1016/j.neuroimage.2011.01.084

[107] Pierce K, Müller RA, Ambrose J, Allen G, Courchesne E (2001) Face processing occurs outside the fusiform ‘face area’ in autism: evidence from functional MRI. Brain 124: 2059–2073. 11571222 10.1093/brain/124.10.2059

[108] Schultz RT, Gauthier I, Klin A, Fulbright RK, Anderson AW, et al (2000) Abnormal ventral temporal cortical activity during face discrimination among individuals with autism and Asperger syndrome. Arch Gen Psychiatry 57: 331–340. 10768694 10.1001/archpsyc.57.4.331

[109] Kleinhans NM, Richards T, Sterling L, Stegbauer KC, Mahurin R, et al (2008) Abnormal functional connectivity in autism spectrum disorders during face processing. Brain 131: 1000–1012. 18234695 10.1093/brain/awm334

[110] Hadjikhani N, Joseph RM, Snyder J, Chabris CF, Clark J, et al (2004) Activation of the fusiform gyrus when individuals with autism spectrum disorder view faces. Neuroimage 22: 1141–1150. 15219586 10.1016/j.neuroimage.2004.03.025

[111] van Kooten IA, Palmen SJ, von Cappeln P, Steinbusch HW, Korr H, et al (2008) Neurons in the fusiform gyrus are fewer and smaller in autism. Brain 131: 987–999. 18332073 10.1093/brain/awn033

[112] Jiao Y, Chen R, Ke X, Chu K, Lu Z, et al (2010) Predictive models of autism spectrum disorder based on brain regional cortical thickness. Neuroimage 50: 589–599. 20026220 10.1016/j.neuroimage.2009.12.047 PMC2823830

[113] Ke X, Hong S, Tang T, Zou B, Li H, et al (2008) Voxel-based morphometry study on brain structure in children with high-functioning autism. Neuroreport 19: 921–925. 18520994 10.1097/WNR.0b013e328300edf3

[114] Uddin LQ, Menon V, Young CB, Ryali S, Chen T, et al (2011) Multivariate searchlight classification of structural magnetic resonance imaging in children and adolescents with autism. Biol Psychiatry 70: 833–841. 21890111 10.1016/j.biopsych.2011.07.014 PMC3191298

