# Supplementary material for: Autistic Traits in Neurotypical Adults: Correlates of Graph Theoretical Functional Network Topology and White Matter Anisotropy Patterns
Source: PLoS One. 2013 Apr 5;8(4):e60982. doi: 10.1371/journal.pone.0060982 (PMC3618514; doi:10.1371/journal.pone.0060982)
Supplement: Table S1 — Description of the custom brain region system used for creating network nodes and regions of the diffusion anisotropy analysis. (DOCX) [file pone.0060982.s001.docx]

| **Number** | **Long name** | **Side** | **Abbreviated name** | **Original atlas** |
| --- | --- | --- | --- | --- |
| 1 | Cerebellum, Crus I. | Left | l-CerCrI | UCL Probabilistic Cerebellar Atlas |
| 2 | Cerebellum, Crus II. | Left | l-CerCrII | UCL Probabilistic Cerebellar Atlas |
| 3 | Cerebellum, Lobules I-IV. | Left | l-CerIIV | UCL Probabilistic Cerebellar Atlas |
| 4 | Cerebellum, Lobule IX. | Left | l-CerIX | UCL Probabilistic Cerebellar Atlas |
| 5 | Cerebellum, Lobule V. | Left | l-CerV | UCL Probabilistic Cerebellar Atlas |
| 6 | Cerebellum, Lobule VI. | Left | l-CerVI | UCL Probabilistic Cerebellar Atlas |
| 7 | Cerebellum, Lobule VIII/a. | Left | l-CerVIIIa | UCL Probabilistic Cerebellar Atlas |
| 8 | Cerebellum, Lobule VIII/b. | Left | l-CerVIIIb | UCL Probabilistic Cerebellar Atlas |
| 9 | Cerebellum, Lobule VII/b. | Left | l-CerVIIb | UCL Probabilistic Cerebellar Atlas |
| 10 | Cerebellum, Lobule X. | Left | l-CerX | UCL Probabilistic Cerebellar Atlas |
| 11 | Cerebellum, Vermis, X. | Midline | l-CverX | UCL Probabilistic Cerebellar Atlas |
| 12 | Cerebellum, Vermis, VI. | Midline | l-CverVI | UCL Probabilistic Cerebellar Atlas |
| 13 | Cerebellum, Vermis, VIII/b. | Midline | l-CverVIIIb | UCL Probabilistic Cerebellar Atlas |
| 14 | Cerebellum, Vermis, VII/b. | Midline | l-CverVIIb | UCL Probabilistic Cerebellar Atlas |
| 15 | Accumbens nucleus | Left | l-ACC | Harvard-Oxford Subcortical Atlas |
| 16 | Caudate nucleus | Left | l-CAUD | Harvard-Oxford Subcortical Atlas |
| 17 | Cingulate gyrus, anterior part | Left | l-CingGyAnt | Harvard-Oxford Cortical Atlas |
| 18 | Frontal medial cortex | Left | l-FrMedC | Harvard-Oxford Cortical Atlas |
| 19 | Frontal opercular cortex | Left | l-FrOpC | Harvard-Oxford Cortical Atlas |
| 20 | Frontal orbital cortex | Left | l-FrOrbC | Harvard-Oxford Cortical Atlas |
| 21 | Frontal pole | Left | l-FrP | Harvard-Oxford Cortical Atlas |
| 22 | Inferior Frontal Gyrus, pars opercularis | Left | l-InfFrGyOp | Harvard-Oxford Cortical Atlas |
| 23 | Inferior Frontal Gyrus, pars triangularis | Left | l-InfFrGyTri | Harvard-Oxford Cortical Atlas |
| 24 | Juxtapositional Lobule Cortex (formerly Supplementary Motor Cortex) | Left | l-JuxtLC | Harvard-Oxford Cortical Atlas |
| 25 | Middle frontal gyrus | Left | l-MiFrGy | Harvard-Oxford Cortical Atlas |
| 26 | Paracingulate gyrus | Left | l-ParCingGy | Harvard-Oxford Cortical Atlas |
| 27 | Precentral gyrus | Left | l-PreCentGy | Harvard-Oxford Cortical Atlas |
| 28 | Subcallosal cortex | Left | l-SubCallC | Harvard-Oxford Cortical Atlas |
| 29 | Superior frontal gyrus | Left | l-SupFrGy | Harvard-Oxford Cortical Atlas |
| 30 | Insular cortex | Left | l-InsC | Harvard-Oxford Cortical Atlas |
| 31 | Cuneal cortex | Left | l-CunC | Harvard-Oxford Cortical Atlas |
| 32 | Intracalcarine cortex | Left | l-IntCalC | Harvard-Oxford Cortical Atlas |
| 33 | Lateral Occipital Cortex, inferior division | Left | l-LatOccCInf | Harvard-Oxford Cortical Atlas |
| 34 | Lingual gyrus | Left | l-LingGy | Harvard-Oxford Cortical Atlas |
| 35 | Occipital Fusiform Gyrus | Left | l-OccFusGy | Harvard-Oxford Cortical Atlas |
| 36 | Occipital pole | Left | l-OccP | Harvard-Oxford Cortical Atlas |
| 37 | Supracalcarine cortex | Left | l-SupCalcC | Harvard-Oxford Cortical Atlas |
| 38 | Angular gyrus | Left | l-AngGy | Harvard-Oxford Cortical Atlas |
| 39 | Central opercular cortex | Left | l-CentOpC | Harvard-Oxford Cortical Atlas |
| 40 | Cingulate gyrus, posterior part | Left | l-CingGyP | Harvard-Oxford Cortical Atlas |
| 41 | Lateral Occipital Cortex, superior division | Left | l-LatOccCS | Harvard-Oxford Cortical Atlas |
| 42 | Parietal operculum cortex | Left | l-ParOpC | Harvard-Oxford Cortical Atlas |
| 43 | Postcentral gyrus | Left | l-PCentGy | Harvard-Oxford Cortical Atlas |
| 44 | Precuneus cortex | Left | l-PreCC | Harvard-Oxford Cortical Atlas |
| 45 | Superior Parietal Lobule | Left | l-SipParL | Harvard-Oxford Cortical Atlas |
| 46 | Supramarginal Gyrus, anterior division | Left | l-SupMGyA | Harvard-Oxford Cortical Atlas |
| 47 | Supramarginal Gyrus, posterior division | Left | l-SupMGyP | Harvard-Oxford Cortical Atlas |
| 48 | Pallidum | Left | l-Pall | Harvard-Oxford Subcortical Atlas |
| 49 | Putamen | Left | l-Put | Harvard-Oxford Subcortical Atlas |
| 50 | Heschl's Gyrus (includes H1 and H2) | Left | l-HschGy | Harvard-Oxford Cortical Atlas |
| 51 | Inferior Temporal Gyrus, anterior division | Left | l-InfTempGyA | Harvard-Oxford Cortical Atlas |
| 52 | Inferior Temporal Gyrus, posterior division | Left | l-InfTempGyP | Harvard-Oxford Cortical Atlas |
| 53 | Inferior Temporal Gyrus, temporooccipital part | Left | l-InfTempGyT | Harvard-Oxford Cortical Atlas |
| 54 | Amygdala | Left | l-Amyg | Harvard-Oxford Subcortical Atlas |
| 55 | Hippocampus | Left | l-Hyp | Harvard-Oxford Subcortical Atlas |
| 56 | Middle Temporal Gyrus, anterior division | Left | l-MidTempGyA | Harvard-Oxford Cortical Atlas |
| 57 | Middle Temporal Gyrus, posterior division | Left | l-MidTempGyP | Harvard-Oxford Cortical Atlas |
| 58 | Middle Temporal Gyrus, temporooccipital part | Left | l-MidTempGyT | Harvard-Oxford Cortical Atlas |
| 59 | Parahippocampal Gyrus, anterior division | Left | l-ParHipGyA | Harvard-Oxford Cortical Atlas |
| 60 | Parahippocampal Gyrus, posterior division | Left | l-ParHipGyP | Harvard-Oxford Cortical Atlas |
| 61 | Planum Polare | Left | l-PlaP | Harvard-Oxford Cortical Atlas |
| 62 | Planum temporale | Left | l-PlaT | Harvard-Oxford Cortical Atlas |
| 63 | Superior Temporal Gyrus, anterior division | Left | l-SupTempGyA | Harvard-Oxford Cortical Atlas |
| 64 | Superior Temporal Gyrus, posterior division | Left | l-SupTempGyP | Harvard-Oxford Cortical Atlas |
| 65 | Temporal Fusiform Cortex, anterior division | Left | l-TempFusCA | Harvard-Oxford Cortical Atlas |
| 66 | Temporal Fusiform Cortex, posterior division | Left | l-TempFusCP | Harvard-Oxford Cortical Atlas |
| 67 | Temporal Occipital Fusiform Cortex | Left | l-TempOccFusC | Harvard-Oxford Cortical Atlas |
| 68 | Temporal pole | Left | l-TempP | Harvard-Oxford Cortical Atlas |
| 69 | Thalamus connection domains - Occipital lobe | Left | l-ThOcc | Oxford thalamic connectivity atlas |
| 70 | Thalamus connection domains - Posterior parietal lobe | Left | l-ThPostPar | Oxford thalamic connectivity atlas |
| 71 | Thalamus connection domains - Pre-frontal cortex | Left | l-ThPreFr | Oxford thalamic connectivity atlas |
| 72 | Thalamus connection domains - Pre-motor cortex | Left | l-ThPreM | Oxford thalamic connectivity atlas |
| 73 | Thalamus connection domains - Primary motor cortex | Left | l-ThPriM | Oxford thalamic connectivity atlas |
| 74 | Thalamus connection domains - Precentral gyrus | Left | l-ThSen | Oxford thalamic connectivity atlas |
| 75 | Thalamus connection domains - Temporal lobe | Left | l-ThTemp | Oxford thalamic connectivity atlas |
| 76 | Cerebellum, Crus I. | Right | r-CerCrI | UCL Probabilistic Cerebellar Atlas |
| 77 | Cerebellum, Crus II. | Right | r-CerCrII | UCL Probabilistic Cerebellar Atlas |
| 78 | Cerebellum, Lobules I-IV. | Right | r-CerIIV | UCL Probabilistic Cerebellar Atlas |
| 79 | Cerebellum, Lobule IX. | Right | r-CerIX | UCL Probabilistic Cerebellar Atlas |
| 80 | Cerebellum, Lobule V. | Right | r-CerV | UCL Probabilistic Cerebellar Atlas |
| 81 | Cerebellum, Lobule VI. | Right | r-CerVI | UCL Probabilistic Cerebellar Atlas |
| 82 | Cerebellum, Lobule VIII/a. | Right | r-CerVIIIa | UCL Probabilistic Cerebellar Atlas |
| 83 | Cerebellum, Lobule VIII/b. | Right | r-CerVIIIb | UCL Probabilistic Cerebellar Atlas |
| 84 | Cerebellum, Lobule VII/b. | Right | r-CerVIIb | UCL Probabilistic Cerebellar Atlas |
| 85 | Cerebellum, Lobule X. | Right | r-CerX | UCL Probabilistic Cerebellar Atlas |
| 86 | Cerebellum, Vermis, II. | Midline | r-CverII | UCL Probabilistic Cerebellar Atlas |
| 87 | Cerebellum, Vermis, VIII/a. | Midline | r-CverVIIIa | UCL Probabilistic Cerebellar Atlas |
| 88 | Cerebellum, Vermis, X. | Midline | r-CverX | UCL Probabilistic Cerebellar Atlas |
| 89 | Accumbens nucleus | Right | r-ACC | Harvard-Oxford Subcortical Atlas |
| 90 | Caudate nucleus | Right | r-CAUD | Harvard-Oxford Subcortical Atlas |
| 91 | Cingulate gyrus, anterior part | Right | r-CingGyAnt | Harvard-Oxford Cortical Atlas |
| 92 | Frontal medial cortex | Right | r-FrMedC | Harvard-Oxford Cortical Atlas |
| 93 | Frontal opercular cortex | Right | r-FrOpC | Harvard-Oxford Cortical Atlas |
| 94 | Frontal orbital cortex | Right | r-FrOrbC | Harvard-Oxford Cortical Atlas |
| 95 | Frontal pole | Right | r-FrP | Harvard-Oxford Cortical Atlas |
| 96 | Inferior Frontal Gyrus, pars opercularis | Right | r-InfFrGyOp | Harvard-Oxford Cortical Atlas |
| 97 | Inferior Frontal Gyrus, pars triangularis | Right | r-InfFrGyTri | Harvard-Oxford Cortical Atlas |
| 98 | Juxtapositional Lobule Cortex (formerly Supplementary Motor Cortex) | Right | r-JuxtLC | Harvard-Oxford Cortical Atlas |
| 99 | Middle frontal gyrus | Right | r-MiFrGy | Harvard-Oxford Cortical Atlas |
| 100 | Paracingulate gyrus | Right | r-ParCingGy | Harvard-Oxford Cortical Atlas |
| 101 | Precentral gyrus | Right | r-PreCentGy | Harvard-Oxford Cortical Atlas |
| 102 | Subcallosal cortex | Right | r-SubCallC | Harvard-Oxford Cortical Atlas |
| 103 | Superior frontal gyrus | Right | r-SupFrGy | Harvard-Oxford Cortical Atlas |
| 104 | Insular cortex | Right | r-InsC | Harvard-Oxford Cortical Atlas |
| 105 | Cuneal cortex | Right | r-CunC | Harvard-Oxford Cortical Atlas |
| 106 | Intracalcarine cortex | Right | r-IntCalC | Harvard-Oxford Cortical Atlas |
| 107 | Lateral Occipital Cortex, inferior division | Right | r-LatOccCInf | Harvard-Oxford Cortical Atlas |
| 108 | Lingual gyrus | Right | r-LingGy | Harvard-Oxford Cortical Atlas |
| 109 | Occipital Fusiform Gyrus | Right | r-OccFusGy | Harvard-Oxford Cortical Atlas |
| 110 | Occipital pole | Right | r-OccP | Harvard-Oxford Cortical Atlas |
| 111 | Supracalcarine cortex | Right | r-SupCalcC | Harvard-Oxford Cortical Atlas |
| 112 | Angular gyrus | Right | r-AngGy | Harvard-Oxford Cortical Atlas |
| 113 | Central opercular cortex | Right | r-CentOpC | Harvard-Oxford Cortical Atlas |
| 114 | Cingulate gyrus, posterior part | Right | r-CingGyP | Harvard-Oxford Cortical Atlas |
| 115 | Lateral Occipital Cortex, superior division | Right | r-LatOccCS | Harvard-Oxford Cortical Atlas |
| 116 | Parietal operculum cortex | Right | r-ParOpC | Harvard-Oxford Cortical Atlas |
| 117 | Postcentral gyrus | Right | r-PCentGy | Harvard-Oxford Cortical Atlas |
| 118 | Precuneus cortex | Right | r-PreCC | Harvard-Oxford Cortical Atlas |
| 119 | Superior Parietal Lobule | Right | r-SipParL | Harvard-Oxford Cortical Atlas |
| 120 | Supramarginal Gyrus, anterior division | Right | r-SupMGyA | Harvard-Oxford Cortical Atlas |
| 121 | Supramarginal Gyrus, posterior division | Right | r-SupMGyP | Harvard-Oxford Cortical Atlas |
| 122 | Pallidum | Right | r-Pall | Harvard-Oxford Subcortical Atlas |
| 123 | Putamen | Right | r-Put | Harvard-Oxford Subcortical Atlas |
| 124 | Heschl's Gyrus (includes H1 and H2) | Right | r-HschGy | Harvard-Oxford Cortical Atlas |
| 125 | Inferior Temporal Gyrus, anterior division | Right | r-InfTempGyA | Harvard-Oxford Cortical Atlas |
| 126 | Inferior Temporal Gyrus, posterior division | Right | r-InfTempGyP | Harvard-Oxford Cortical Atlas |
| 127 | Inferior Temporal Gyrus, temporooccipital part | Right | r-InfTempGyT | Harvard-Oxford Cortical Atlas |
| 128 | Amygdala | Right | r-Amyg | Harvard-Oxford Subcortical Atlas |
| 129 | Hippocampus | Right | r-Hyp | Harvard-Oxford Subcortical Atlas |
| 130 | Middle Temporal Gyrus, anterior division | Right | r-MidTempGyA | Harvard-Oxford Cortical Atlas |
| 131 | Middle Temporal Gyrus, posterior division | Right | r-MidTempGyP | Harvard-Oxford Cortical Atlas |
| 132 | Middle Temporal Gyrus, temporooccipital part | Right | r-MidTempGyT | Harvard-Oxford Cortical Atlas |
| 133 | Parahippocampal Gyrus, anterior division | Right | r-ParHipGyA | Harvard-Oxford Cortical Atlas |
| 134 | Parahippocampal Gyrus, posterior division | Right | r-ParHipGyP | Harvard-Oxford Cortical Atlas |
| 135 | Planum Polare | Right | r-PlaP | Harvard-Oxford Cortical Atlas |
| 136 | Planum temporale | Right | r-PlaT | Harvard-Oxford Cortical Atlas |
| 137 | Superior Temporal Gyrus, anterior division | Right | r-SupTempGyA | Harvard-Oxford Cortical Atlas |
| 138 | Superior Temporal Gyrus, posterior division | Right | r-SupTempGyP | Harvard-Oxford Cortical Atlas |
| 139 | Temporal Fusiform Cortex, anterior division | Right | r-TempFusCA | Harvard-Oxford Cortical Atlas |
| 140 | Temporal Fusiform Cortex, posterior division | Right | r-TempFusCP | Harvard-Oxford Cortical Atlas |
| 141 | Temporal Occipital Fusiform Cortex | Right | r-TempOccFusC | Harvard-Oxford Cortical Atlas |
| 142 | Temporal pole | Right | r-TempP | Harvard-Oxford Cortical Atlas |
| 143 | Thalamus connection domains - Occipital lobe | Right | r-ThOcc | Oxford thalamic connectivity atlas |
| 144 | Thalamus connection domains - Posterior parietal lobe | Right | r-ThTemp | Oxford thalamic connectivity atlas |
| 145 | Thalamus connection domains - Pre-frontal cortex | Right | r-ThS | Oxford thalamic connectivity atlas |
| 146 | Thalamus connection domains - Pre-motor cortex | Right | r-ThPostPar | Oxford thalamic connectivity atlas |
| 147 | Thalamus connection domains - Primary motor cortex | Right | r-ThPreFr | Oxford thalamic connectivity atlas |
| 148 | Thalamus connection domains - Precentral gyrus | Right | r-ThPreM | Oxford thalamic connectivity atlas |
| 149 | Thalamus connection domains - Temporal lobe | Right | r-ThPriM | Oxford thalamic connectivity atlas |
