# Supplementary material for: Autistic Traits in Neurotypical Adults: Correlates of Graph Theoretical Functional Network Topology and White Matter Anisotropy Patterns
Source: PLoS One. 2013 Apr 5;8(4):e60982. doi: 10.1371/journal.pone.0060982 (PMC3618514; doi:10.1371/journal.pone.0060982)
Supplement: Document S1 — Pseudo code of cost integration method used for calculating the global and regional graph theoretical network descriptors. (PDF) [file pone.0060982.s003.pdf]

Algorithm 1 Pseudo code of cost integration method

procedure COSTINTEGRATION($WeightedNetwork, CostRange, Repetition$)
    $r \leftarrow Repetition$
    while $r \neq 0$ **do**
        $cost \leftarrow random\ value\ from\ CostRange$
        $BinaryNetwork \leftarrow WeightedNetwork(cost)$
        $k_i^{cost} \leftarrow all\ nodes\ of\ BinaryNetwork$
        $e_i^{cost} \leftarrow all\ nodes\ of\ BinaryNetwork$
        $Eg^{cost} \leftarrow BinaryNetwork$
        $El^{cost} \leftarrow BinaryNetwork$
        $r \leftarrow r - 1$
    end while
    $k_i^* \leftarrow \sum k_i^{cost}$
    $e_i^* \leftarrow \sum e_i^{cost}$
    $Eg^* \leftarrow \sum Eg^{cost}$
    $El^* \leftarrow \sum El^{cost}$
    return k_i^*, e_i^*, Eg^*, El^*
end procedure
