# Supplementary material for: Autistic Traits in Neurotypical Adults: Correlates of Graph Theoretical Functional Network Topology and White Matter Anisotropy Patterns
Source: PLoS One. 2013 Apr 5;8(4):e60982. doi: 10.1371/journal.pone.0060982 (PMC3618514; doi:10.1371/journal.pone.0060982)
Supplement: Document S2 — Scripts used in SPSS to perform GLM analysis and false discovery rate adjustment. (DOCX) [file pone.0060982.s004.docx]

**Supplementary Document 1. Scripts used in SPSS to perform GLM analysis**

**GLM Regression Model 1. SPSS syntax.**

GLM (list of variables to predict) WITH tot_r assq age gender subj
/METHOD=SSTYPE(3)
/INTERCEPT=INCLUDE
/LMATRIX
tot_r -1 assq -1 intercept 0 age 0 gender 0 subj 0;
/PRINT = TEST(LMATRIX) PARAMETER ETASQ
/CRITERIA=ALPHA(.05)
/DESIGN.

**GLM Regression Model 2. SPSS syntax.**

GLM (list of variables to predict) WITH tot_r assq age gender subj verbalIQ
/METHOD=SSTYPE(3)
/INTERCEPT=INCLUDE
/LMATRIX
tot_r -1 assq -1 intercept 0 gender 0 subj 0 verbalIQ 0;
/PRINT = TEST(LMATRIX) PARAMETER ETASQ
/CRITERIA=ALPHA(.05)
/DESIGN.

**False discovery rate adjustment, Benjamini-Hochberg procedure. SPSS syntax.**

sort cases by p (a).
compute i=$casenum.
sort cases by i (d).
compute q=.05.
compute m=max(i,lag(m)).
compute crit=q*i/(m*1).
compute test=(p le crit).
compute test=max(test,lag(test)).
execute.
formats i m test(f8.0) q (f8.2) crit(f8.6).
value labels test 1 'Significant' 0 'Not Significant'.
